# Vertebrobasilar artery geometry and quantitative high-resolution vessel wall MRI for stratifying vulnerable plaques in young and middle-aged adults with stroke risk factors

**DOI:** 10.3389/fmed.2026.1914044

**Published:** 2026-08-12

**Authors:** Xin Liu, Bo Zheng, Lei Wang, Ye Zheng, Wenbin Luo, Gen Yan

**Affiliations:** 1 Department of Radiology, The Second Affiliated Hospital of Xiamen Medical College, Xiamen, Fujian, China; 2 Department of Urology, The Fifth Hospital of Xiamen City, Xiamen, Fujian, China

**Keywords:** high-resolution vessel wall MRI, intracranial atherosclerosis, vascular geometry, vertebrobasilar artery, vulnerable plaque

## Abstract

**Objective:**

To investigate cross-sectional associations of vertebrobasilar arterial geometry and quantitative high-resolution vessel-wall imaging (HR-VWI) features with an HR-VWI-defined vulnerable plaque phenotype in individuals with stroke risk factors, and to evaluate exploratory imaging models for plaque stratification.

**Methods:**

This single-center cross-sectional study included 174 patients aged 18–59 years with stroke risk factors and measurable vertebrobasilar atherosclerotic plaques on HR-VWI. Patients were classified as having vulnerable or non-vulnerable plaques according to HR-VWI features: marked plaque enhancement, intraplaque hemorrhage, irregular plaque surface, or marked signal heterogeneity. Vertebrobasilar geometric parameters and quantitative plaque measurements were assessed. Age and body mass index (BMI) were included as covariates. Logistic regression and receiver operating characteristic analyses identified imaging factors associated with vulnerable plaque phenotype and compared age-and-BMI, age-and-BMI plus geometry, age-and-BMI plus quantitative HR-VWI, and combined imaging models.

**Results:**

Sixty-nine patients had vulnerable plaques and 105 had non-vulnerable plaques. Vulnerable plaques were associated with greater basilar artery curvature index, larger vertebrobasilar junction angle, greater bilateral vertebral artery diameter difference, greater maximum basilar artery deviation, higher plaque burden, larger wall area, greater percentage area stenosis, and higher remodeling index. In the exploratory multivariable model, plaque burden, percentage area stenosis, remodeling index, and maximum basilar artery deviation remained associated with vulnerable plaque phenotype after adjustment for age and BMI. Development-cohort AUCs were 0.637 for age-and-BMI, 0.774 for age-and-BMI plus geometry, 0.883 for age-and-BMI plus quantitative HR-VWI, and 0.903 for combined imaging. The difference between combined imaging and quantitative HR-VWI was not significant (ΔAUC = 0.020; *P* = 0.162). At optimal thresholds, the combined model had higher specificity (89.52% vs. 76.19%) but lower sensitivity (78.26% vs. 86.96%).

**Conclusion:**

Plaque burden, percentage area stenosis, remodeling index, and maximum basilar artery deviation were associated with the HR-VWI-defined vulnerable plaque phenotype after adjustment for age and BMI. Although combined imaging had the highest numerical AUC, adding vertebrobasilar geometry did not significantly improve discrimination beyond quantitative HR-VWI. Because predictors and phenotype were assessed during the same examination, these AUCs reflect within-examination classification rather than prediction of an independent biological or clinical endpoint. These exploratory findings should not be interpreted as predicting future posterior-circulation ischemic events.

## 1 Introduction

Stroke remains a leading cause of mortality and long-term disability worldwide, with ischemic stroke representing the predominant pathological subtype. Although stroke has traditionally been regarded as a disease of older adults, its incidence and clinical impact in young and middle-aged populations have increased substantially in recent years, largely owing to the earlier onset of hypertension, dyslipidemia, diabetes mellitus, obesity, smoking exposure, and other modifiable vascular risk factors (1–3). Compared with elderly patients, young and middle-aged individuals who experience ischemic stroke often face a longer period of neurological impairment, reduced work capacity, psychological burden, and socioeconomic loss. Therefore, improved imaging characterization of potentially high-risk vascular lesions is particularly relevant in this population (4, 5).

Posterior circulation ischemic stroke accounts for approximately one-fifth to one-quarter of all ischemic strokes and is frequently associated with lesions involving the vertebral arteries, vertebrobasilar junction, basilar artery, brainstem, cerebellum, thalamus, and posterior cerebral territories (6, 7). Owing to the compact distribution of vital neurological structures in the brainstem and cerebellum, posterior circulation ischemia may present with nonspecific or fluctuating symptoms, while still carrying a substantial risk of clinical deterioration, recurrence, and unfavorable outcomes (8). Intracranial atherosclerotic disease of the vertebrobasilar system is one of the principal mechanisms underlying posterior circulation ischemia. However, the biological behavior of vertebrobasilar atherosclerotic plaques cannot be fully characterized by the degree of luminal stenosis alone, because plaque activity, remodeling pattern, surface morphology, intraplaque components, and local hemodynamic stress may all contribute to lesion instability and subsequent ischemic events (9, 10).

The vertebrobasilar arterial system has distinctive anatomical and hemodynamic characteristics. Blood flow entering the basilar artery is determined by bilateral vertebral artery inflow, vertebral artery dominance, diameter asymmetry, confluence geometry, vertebrobasilar junction angle, basilar artery curvature, vessel length, and lateral deviation. These geometric features may alter local flow patterns, wall shear stress, oscillatory shear index, pressure gradients, and particle residence time along the posterior circulation arterial wall (10–12). Regions exposed to disturbed or low wall shear stress are susceptible to endothelial dysfunction, increased permeability, inflammatory cell recruitment, lipid accumulation, and extracellular matrix remodeling, thereby creating a biomechanical environment conducive to plaque initiation and progression. Conversely, complex curvature and asymmetric inflow may generate focal mechanical loading and spatially heterogeneous shear stress, potentially influencing plaque eccentricity, remodeling direction, enhancement behavior, and vulnerability. Thus, vertebrobasilar geometry may represent more than an anatomical descriptor and may be associated with local plaque phenotype through its influence on regional hemodynamics.

Conventional vascular imaging modalities, including computed tomography angiography, time-of-flight magnetic resonance angiography, and digital subtraction angiography, primarily evaluate the arterial lumen and are therefore limited in their ability to characterize the vessel wall. This lumen-centered approach may underestimate disease burden, particularly in plaques with positive remodeling or mild-to-moderate stenosis, where the arterial lumen can be relatively preserved despite substantial wall pathology. In contrast, high-resolution vessel wall magnetic resonance imaging allows direct visualization of the intracranial arterial wall and provides both qualitative and quantitative plaque information. Parameters such as plaque burden, wall area, lumen area, percentage area stenosis, remodeling index, plaque enhancement, surface irregularity, eccentricity, signal heterogeneity, and intraplaque hemorrhage have been associated with plaque vulnerability and symptomatic intracranial atherosclerosis (13–16). Therefore, high-resolution MRI offers a more comprehensive imaging framework for evaluating posterior circulation atherosclerosis than conventional luminal imaging alone.

Previous studies have generally evaluated vertebrobasilar geometry and plaque-wall characteristics separately, and it remains uncertain whether vessel-level geometry provides information complementary to quantitative plaque measurements. Qualitative high-risk plaque features can be directly identified on HR-VWI and remain the primary basis for imaging interpretation. Therefore, the purpose of the present multivariable analysis was not to replace direct qualitative HR-VWI assessment or to predict future ischemic events. Instead, we investigated whether objective quantitative plaque metrics that were not components of the vulnerability definition could classify an HR-VWI-defined vulnerable plaque phenotype and whether incorporation of vertebrobasilar geometry provided incremental discriminatory information beyond local plaque measurements. A combined assessment of vascular geometry and plaque characteristics may provide a more mechanistically informed framework for cross-sectional imaging-based stratification of vertebrobasilar plaque phenotypes.

## 2 Materials and methods

### 2.1 Study design and ethical approval

This single-center, cross-sectional imaging study included consecutive inpatients aged 18–59 years who underwent head and neck magnetic resonance angiography and vertebrobasilar high-resolution vessel-wall MRI at our institution between February 2024 and January 2026. The cohort was not restricted to stroke-naïve individuals; a previous transient ischemic attack or ischemic stroke was permitted and was recorded as a baseline clinical characteristic.

Because all vertebrobasilar geometric parameters, quantitative plaque measurements, and plaque-vulnerability classifications were assessed from the same index imaging examination, the study was designed as a cross-sectional imaging analysis. The primary outcome was the presence of vulnerable plaque on high-resolution vessel wall MRI.

The study was approved by the Ethics Committee of The Second Affiliated Hospital of Xiamen Medical College Approval No 2021050. Written informed consent was obtained from all participants.

### 2.2 Study population

Patients were screened from the institutional radiology database, picture archiving and communication system, and electronic medical record system. Young and middle-aged individuals were defined as patients aged 18–59 years. Patients were considered at high risk of stroke if they had at least one established cerebrovascular risk factor, including hypertension, diabetes mellitus, dyslipidemia, smoking, obesity, previous transient ischemic attack or ischemic stroke, family history of stroke, hyperhomocysteinemia, or imaging evidence of intracranial or extracranial atherosclerosis.

The inclusion criteria were as follows: (1) age between 18 and 59 years; (2) presence of one or more stroke-related vascular risk factors; (3) availability of head and neck MRA and vertebrobasilar high-resolution vessel wall MRI; (4) MRI evidence of at least one measurable, nonocclusive atherosclerotic plaque in the V4 segment of either vertebral artery, the vertebrobasilar junction, or the basilar artery. A measurable plaque was defined as focal or eccentric arterial-wall thickening on multiplanar HR-VWI for which the plaque margin, lumen boundary, outer vessel-wall boundary, and an appropriate reference arterial segment could be clearly delineated, thereby permitting calculation of plaque burden, percentage area stenosis, and remodeling index. No minimum luminal stenosis threshold was required. Accordingly, plaques with 0% to < 100% area stenosis were eligible, whereas complete arterial occlusions were excluded because the residual lumen and plaque-site lumen area could not be reliably measured; (5) sufficient image quality for delineation of the lumen boundary, outer vessel wall boundary, plaque extent, and enhancement characteristics; and (6) availability of complete clinical, laboratory, and imaging data required for the primary analyses.

The exclusion criteria were as follows: (1) non-atherosclerotic intracranial or extracranial vascular disease, including arteritis, moyamoya disease, arterial dissection, vascular malformation, aneurysm, or suspected dissection; (2) definite cardioembolic source, hematological disease, malignant tumor, severe infection, or active autoimmune disease; (3) previous stent implantation, balloon angioplasty, vascular reconstruction, or other interventional treatment involving the vertebral or basilar artery; (4) contraindication to MRI or inability to complete the examination because of claustrophobia, severe involuntary movement, or poor cooperation; (5) contraindication to contrast-enhanced MRI, severe renal dysfunction, or history of gadolinium-based contrast agent allergy; (6) severe motion artifacts, flow artifacts, or susceptibility artifacts affecting vessel wall or plaque assessment; and (7) missing key clinical variables or core imaging indicators.

After application of the inclusion and exclusion criteria, 174 patients were included in the final analysis. According to high-resolution vessel wall MRI plaque features, patients were classified into a vulnerable plaque group and a non-vulnerable plaque group.

The sampling frame therefore represented an inpatient, HR-VWI-referred, plaque-positive population and was not intended to represent all individuals with intracranial atherosclerosis in the general or outpatient population.

### 2.3 Clinical and laboratory data collection

Data completeness was assessed for all variables used in the primary analyses before statistical analysis. Essential variables included the demographic and clinical characteristics reported in Table 1, vertebrobasilar geometric measurements, quantitative and qualitative HR-VWI variables, plaque-phenotype classification, and all variables entered into the exploratory imaging models. Patients with missing essential clinical information or incomplete, non-diagnostic, or non-measurable imaging data were excluded during cohort screening. Consequently, the final analytical cohort of 174 patients had complete data for all variables included in the primary analyses, corresponding to a missingness rate of 0/174 (0%).

**TABLE 1:** Baseline characteristics of patients according to plaque vulnerability.

Characteristic	Vulnerable plaque group (*n* = 69)	Non-vulnerable plaque group (*n* = 105)	Standardized difference	*P*-value
Age, years	48.4 ± 7.0	45.9 ± 7.6	0.34	0.032
Male sex	49/69 (71.0)	65/105 (61.9)	0.19	0.216
Body mass index, kg/m^2^	25.9 ± 3.1	24.8 ± 3.0	0.36	0.021
Hypertension	46/69 (66.7)	57/105 (54.3)	0.26	0.104
Diabetes mellitus	23/69 (33.3)	24/105 (22.9)	0.23	0.128
Dyslipidemia	50/69 (72.5)	65/105 (61.9)	0.23	0.150
Current smoking	40/69 (58.0)	50/105 (47.6)	0.21	0.181
Alcohol consumption	27/69 (39.1)	33/105 (31.4)	0.16	0.296
Previous TIA or ischemic stroke	19/69 (27.5)	18/105 (17.1)	0.25	0.101

Values are presented as mean ± SD or n/N (%). Standardized differences were used to quantify between-group imbalance. TIA, transient ischemic attack.

Because all included participants were inpatients, the primary admission diagnosis was extracted from the electronic medical record and defined as the first-listed principal discharge diagnosis representing the main reason for hospitalization. The diagnoses were grouped into mutually exclusive clinical categories, and their frequencies and constituent ratios are reported in Supplementary Table 3.

### 2.4 MRI acquisition protocol

All examinations were performed using a 3.0-T MRI scanner GE Discovery 750 3.0T with a dedicated head-neck coil. Patients were examined in the supine position with head fixation to reduce motion. Before scanning, patients were instructed to avoid swallowing, coughing, and head or neck movement as much as possible during image acquisition.

The conventional brain MRI protocol included T1-weighted imaging, T2-weighted imaging, T2-fluid-attenuated inversion recovery imaging, diffusion-weighted imaging, and apparent diffusion coefficient mapping. These sequences were used to evaluate the presence of acute or chronic ischemic lesions. However, DWI-positive posterior-circulation infarction, culprit-plaque attribution, and symptom–plaque territorial concordance were not prespecified study variables and were not used to define the HR-VWI plaque phenotype or develop the imaging classification models. The conventional brain MRI sequences were acquired as part of routine clinical imaging rather than as an independently adjudicated external outcome for the present cross-sectional analysis. Three-dimensional time-of-flight MRA was performed to assess luminal morphology of the vertebral arteries, vertebrobasilar junction, basilar artery, and major intracranial arteries, and served as the anatomical reference for vascular geometry assessment.

The HR-VWI acquisition covered the bilateral V4 segments of the vertebral arteries, the vertebrobasilar junction, and the entire basilar artery. Black-blood vessel-wall imaging was performed using three-dimensional variable-flip-angle turbo spin-echo sequences with sampling perfection with application-optimized contrasts using different flip-angle evolution (SPACE). All HR-VWI sequences were acquired with a field of view of 160 × 160 mm, a matrix of 320 × 320, a reconstructed voxel size of 0.50 × 0.50 × 0.60 mm, a slice thickness of 0.60 mm, no interslice gap, and one excitation.

The precontrast black-blood T1-weighted sequence was acquired with a repetition time/echo time of 900/15 ms and an acquisition time of approximately 5 min 30 s. The black-blood T2-weighted sequence used a repetition time/echo time of 2,500/90 ms and an acquisition time of approximately 5 min 45 s, while the proton-density-weighted sequence used a repetition time/echo time of 2,000/30 ms and an acquisition time of approximately 5 min 20 s. Contrast-enhanced black-blood T1-weighted HR-VWI was acquired using the same spatial resolution and a repetition time/echo time of 900/15 ms. Gadobutrol was administered intravenously at 0.1 mmol/kg body weight, followed by a 20-mL normal-saline flush, and postcontrast imaging was initiated approximately 5 min after contrast administration. The postcontrast T1-weighted acquisition time was approximately 5 min 30 s.

The acquisition protocol remained unchanged throughout the study period to minimize technical variability. Complete acquisition parameters for the conventional brain MRI, three-dimensional time-of-flight MRA, and HR-VWI sequences are provided in Supplementary Table 1, while the detailed contrast-enhanced HR-VWI protocol is provided in Supplementary Table 2.

### 2.5 Image post-processing and quality assessment

All imaging data were exported from the picture archiving and communication system and analyzed on a dedicated imaging workstation. Multiplanar reconstruction was performed when necessary to obtain cross-sectional images perpendicular to the long axis of the target artery. Image quality was assessed before quantitative analysis. Images were considered eligible when the lumen boundary, outer wall boundary, plaque margin, and enhancement characteristics could be clearly evaluated. Images with severe motion artifacts, flow artifacts, susceptibility artifacts, incomplete coverage, or poor vessel wall contrast were excluded.

All HR-VWI and TOF-MRA examinations were anonymized before image analysis. Two radiologists experienced in neurovascular MRI independently evaluated the images on the same dedicated workstation. Both readers were blinded to the patients’ clinical characteristics, laboratory findings, previous imaging reports, group assignments, and statistical results, and neither reader had access to the other reader’s assessments.

Each reader independently measured the vertebrobasilar geometric parameters, including basilar artery centerline length, straight-line length, curvature index, vertebrobasilar junction angle, basilar artery diameter, bilateral vertebral artery diameter difference, and maximum basilar artery deviation. Each reader also independently delineated the lumen and outer vessel-wall boundaries and calculated lumen area, total vessel area, wall area, plaque burden, percentage area stenosis, and remodeling index. Qualitative variables—including plaque distribution, enhancement grade, surface morphology, intraplaque hemorrhage, signal heterogeneity, remodeling category, vertebral artery dominance, basilar artery bending grade, and the HR-VWI-defined vulnerable plaque phenotype—were assessed independently.

Interobserver reproducibility was calculated using the original independent readings before consensus resolution. Disagreements in qualitative assessments were resolved by consensus after completion of the independent evaluations; unresolved cases were adjudicated by a senior neuroradiologist. For quantitative parameters, the mean of the two independent reader measurements was used in the primary analyses.

### 2.6 Definition of vulnerable plaque and grouping strategy

An intracranial atherosclerotic plaque was defined as focal or eccentric arterial-wall thickening relative to the adjacent normal-appearing vessel wall, with or without luminal narrowing or outward remodeling, in accordance with expert consensus recommendations for intracranial vessel-wall MRI (17). For the present quantitative analysis, a plaque was considered measurable when its lumen boundary, outer wall boundary, plaque extent, and corresponding reference-segment boundaries could be reliably delineated on multiplanar HR-VWI. Plaque presence was not contingent on a minimum degree of stenosis; nonstenotic plaques and all grades of nonocclusive stenosis were eligible.

A plaque was classified as having an HR-VWI-defined vulnerable plaque phenotype when at least one of the following high-risk imaging features was present: (1) marked plaque enhancement on contrast-enhanced T1-weighted imaging; (2) intraplaque hemorrhage; (3) an irregular plaque surface; or (4) marked intraplaque signal heterogeneity indicating complex plaque composition. Plaques without any of these features were classified as non-vulnerable. These criteria represented an imaging-based classification rather than histopathological confirmation of plaque vulnerability. These imaging criteria were adapted from expert consensus recommendations and representative intracranial plaque studies (17–21).

For patients with multiple plaques, the patient was assigned to the vulnerable plaque group if any plaque fulfilled the vulnerability criteria. In such cases, the vulnerable plaque with the most prominent high-risk imaging features was selected as the index plaque for quantitative analysis. In patients without vulnerable plaque features, the plaque with the largest plaque burden was selected as the index plaque.

### 2.7 Assessment of vertebrobasilar arterial geometry

Vertebrobasilar arterial geometry was assessed on three-dimensional TOF-MRA using centerline-based and multiplanar reconstructions, following previously reported vertebrobasilar morphometric approaches (22, 23). Vertebrobasilar configuration, vertebral artery asymmetry, arterial bending, and basilar artery course have previously been evaluated as geometric factors associated with basilar artery plaque formation and distribution.

Basilar artery centerline length was measured from the vertebrobasilar junction to the basilar artery apex along the vascular centerline. The straight-line length was measured as the Euclidean distance between the same two landmarks. The basilar artery curvature index was calculated as:


$$\mathrm{Basilar}\,\mathrm{artery}\,\mathrm{curvature}\,\mathrm{index}=\frac{\mathrm{centerline}\,\mathrm{length}}{\mathrm{straight}-\mathrm{line}\,\mathrm{length}}$$

A value closer to 1 indicated a relatively straight arterial course, whereas a higher value indicated greater tortuosity.

The vertebrobasilar junction angle was defined as the angle formed by the centerlines of the distal left and right vertebral arteries at their confluence. Basilar artery diameter was measured at the proximal, middle, and distal thirds of the vessel, and the mean of the three measurements was used. The bilateral vertebral artery diameter difference was calculated as the absolute difference between the mean diameters of the left and right V4 segments. Previous vertebrobasilar studies have similarly assessed bilateral vertebral artery diameter asymmetry and its relationship with basilar artery geometry and plaque occurrence (17, 22).

Maximum basilar artery deviation was defined as the greatest perpendicular distance between the basilar artery centerline and the reference line connecting the vertebrobasilar junction with the basilar artery apex. Basilar artery bending was graded visually according to the magnitude of lateral displacement and curvature. Vertebral artery dominance was classified according to side-to-side V4 diameter asymmetry, using the prespecified study criterion.

Vertebral artery dominance was defined as a side-to-side V4 diameter difference of ≥ 0.3 mm; differences below 0.3 mm were classified as no obvious dominance.

For patients with multiple plaques, the participant was assigned to the vulnerable plaque group if at least one plaque fulfilled the vulnerability criteria. In these participants, the vulnerable plaque showing the most prominent high-risk imaging features was selected as the index plaque. In participants without vulnerable plaque features, the plaque with the largest plaque burden was selected as the index plaque. Because the index-plaque selection rule differed according to vulnerability status, the possibility of outcome-dependent selection bias was considered when interpreting the quantitative comparisons and is acknowledged in the Limitations section.

### 2.8 HR-MRI plaque localization and quantitative measurement

Plaques were classified anatomically as basilar artery plaques or V4-segment vertebral artery plaques. Basilar artery plaques were further categorized as proximal, middle, or distal according to their dominant location. V4-segment vertebral artery plaques were recorded as left-sided or right-sided. Plaques involving the vertebrobasilar junction were classified according to the dominant location of the plaque body.

Quantitative plaque measurements were performed on the cross-sectional slice showing the maximum plaque burden or maximal luminal narrowing. The lumen boundary and outer vessel wall boundary were manually delineated. The following parameters were obtained or calculated: lumen area, total vessel area, wall area, plaque burden, percentage area stenosis, and remodeling index.

Wall area was calculated as:


Wall⁢area=total⁢vessel⁢area-lumen⁢area

Plaque burden was calculated as:


Plaque⁢burden=wall⁢area/total⁢vessel⁢area×100%

Percentage area stenosis was calculated relative to the reference lumen area:


Percentage⁢area⁢stenosis=reference⁢lumen⁢area⁢reference

 ⁢lumen⁢area-plaque-site⁢lumen⁢area/reference

  lumenarea×100

A value of 0% indicated no measurable reduction in lumen area relative to the reference segment. Complete occlusion was not eligible because plaque-site lumen area and wall boundaries could not be evaluated according to the prespecified quantitative protocol.

The reference segment was selected as the nearest normal-appearing arterial segment proximal or distal to the plaque, avoiding branch points, severe curvature, and adjacent plaque extension when possible.

The remodeling index was calculated as:


Remodeling⁢index=total⁢vessel⁢area⁢at⁢the⁢plaque

site/total⁢vessel⁢area⁢at⁢the⁢reference⁢segment

A remodeling index > 1.05 was defined as positive remodeling, a remodeling index between 0.95 and 1.05 as no obvious remodeling, and a remodeling index < 0.95 as negative remodeling.

The unit of analysis was the participant, with one index plaque evaluated per participant. An all-plaque lesion-level analysis was not performed because equivalent quantitative measurements were not systematically available for every additional plaque. Furthermore, multiple plaques within the same participant would require clustered statistical methods, such as generalized estimating equations or mixed-effects regression, to account for within-participant correlation.

### 2.9 Qualitative plaque phenotype assessment

Qualitative HR-VWI plaque features were assessed using definitions adapted from the American Society of Neuroradiology expert consensus recommendations and representative intracranial atherosclerotic plaque studies (17–21). Plaque enhancement was evaluated by comparing precontrast and postcontrast T1-weighted vessel-wall images. Enhancement was graded as follows: grade 0, no obvious enhancement, defined as enhancement similar to or lower than that of the adjacent normal arterial wall; grade 1, mild enhancement, defined as enhancement greater than that of the normal arterial wall but lower than that of the pituitary infundibulum; and grade 2, marked enhancement, defined as enhancement comparable to or greater than that of the pituitary infundibulum. Grade 2 enhancement was considered a high-risk imaging feature.

Intraplaque hemorrhage was defined as focal intraplaque hyperintensity on precontrast T1-weighted vessel-wall imaging, consistent with previously published intracranial HR-VWI criteria (19). Plaque distribution was classified as eccentric or concentric according to the circumferential extent of arterial-wall involvement. Plaque surface morphology was classified as regular or irregular; an irregular surface was defined as a visibly non-smooth or discontinuous luminal contour on multiplanar vessel-wall images. The intraplaque signal pattern was classified as homogeneous or heterogeneous/complex according to the presence of visually distinct signal components across the multicontrast vessel-wall sequences.

### 2.10 Exploratory imaging classification model development

The presence of an HR-VWI-defined vulnerable plaque phenotype was used as the dependent variable. Because the predictors and outcome were obtained from the same index imaging examination, the models were developed as exploratory cross-sectional classification models rather than prognostic models of future ischemic events.

Candidate predictors were restricted to prespecified clinical covariates, vertebrobasilar geometric parameters, and objective quantitative HR-VWI plaque measurements. Qualitative features used to define the vulnerable plaque phenotype—including marked enhancement, intraplaque hemorrhage, irregular plaque surface, and complex signal heterogeneity—were excluded a priori to avoid incorporation of outcome-defining variables into the models.

Age and body mass index were prespecified as potential clinical confounders because of their biological relevance and observed baseline imbalance. Both variables were entered as continuous covariates and forced into all multivariable models regardless of their statistical significance. Age was reported per 5-year increase and body mass index per 1-kg/m^2^ increase.

### 2.11 Statistical analysis

Statistical analyses were performed using SPSS version 26.0 (IBM Corp., Armonk, NY, United States) and R version 4.3.0 (R Foundation for Statistical Computing, Vienna, Austria). Continuous variables were first assessed for normality. Normally distributed continuous variables were expressed as mean ± standard deviation and compared using the independent-samples *t*-test. Non-normally distributed continuous variables were expressed as median and interquartile range and compared using the Mann–Whitney U test. Categorical variables were expressed as counts and percentages and compared using the χ^2^ test or Fisher’s exact test, as appropriate. Ordinal variables were compared using rank-sum tests.

Receiver operating characteristic curve analysis was used to evaluate discrimination of the clinical, clinical plus geometry, clinical plus quantitative HR-VWI, and clinical plus combined imaging models. AUCs with 95% confidence intervals were calculated, and optimal probability thresholds were selected by maximizing the Youden index. Because the ROC curves were derived from the same participants, differences between correlated AUCs were evaluated using the nonparametric DeLong test. Prespecified comparisons included each imaging-augmented model versus the clinical model and the clinical plus combined imaging model versus the clinical plus quantitative HR-VWI model. The latter comparison directly evaluated whether incorporation of vertebrobasilar geometric parameters provided incremental discrimination beyond clinical covariates and quantitative HR-VWI measurements. Two-sided *P*-values < 0.05 were considered statistically significant.

Each prespecified candidate predictor was initially assessed using univariable binary logistic regression with the HR-VWI-defined vulnerable plaque phenotype as the dependent variable. Variables with a univariable *P* < 0.10 were considered eligible for entry into the initial multivariable model. Age and body mass index were retained as forced clinical covariates irrespective of their univariable or multivariable *P*-values.

Before multivariable model construction, collinearity among eligible predictors was evaluated using tolerance values and variance inflation factors. A tolerance value ≤ 0.20 or a variance inflation factor ≥ 5 was considered indicative of potentially important multicollinearity. When two or more variables represented overlapping or mathematically related constructs, the variable with greater clinical interpretability, stronger univariable association, and lower collinearity was retained. Thus, continuous and categorical representations of the same measurement were not entered simultaneously, and highly redundant area-derived variables were removed before backward elimination.

The initial multivariable model included all eligible non-collinear predictors together with the forced age and body mass index covariates. Backward likelihood-ratio elimination was then performed. At each step, the non-forced variable with the highest *P*-value was removed when the likelihood-ratio removal probability exceeded 0.10. The entry criterion was *P* < 0.05 and the removal criterion was *P* > 0.10. Age and body mass index were protected from elimination. The elimination procedure continued until no additional non-forced variable met the removal criterion. Adjusted odds ratios, regression coefficients, standard errors, Wald statistics, 95% confidence intervals, and two-sided *P*-values were reported for the final model.

Collinearity diagnostics were repeated in the final model to confirm that all retained predictors had variance inflation factors < 5 and tolerance values > 0.20. Detailed univariable screening and collinearity diagnostics are provided in Supplementary Table 5. The prespecified candidate pool included age, body mass index, basilar artery curvature index, vertebrobasilar junction angle, basilar artery length, mean basilar artery diameter, bilateral vertebral artery diameter difference, maximum basilar artery deviation, plaque burden, wall area, percentage area stenosis, remodeling index, lumen area, and total vessel area. Qualitative plaque features were not considered candidate predictors because the purpose of the model was to assess quantitative imaging information complementary to direct qualitative HR-VWI interpretation.

The linearity of continuous predictors in the logit was assessed before final model fitting. Adjusted odds ratios with 95% confidence intervals were reported. The final adjusted association model contained age, body mass index, plaque burden, percentage area stenosis, remodeling index, and maximum basilar artery deviation. Discriminative performance was assessed separately for the clinical model, clinical plus geometry model, clinical plus quantitative HR-VWI model, and clinical plus combined imaging model.

The age-and-BMI model included age and body mass index. The age-and-BMI plus geometry model additionally included basilar artery curvature index, vertebrobasilar junction angle, bilateral vertebral artery diameter difference, and maximum basilar artery deviation. The age-and-BMI plus quantitative HR-VWI model additionally included plaque burden, wall area, percentage area stenosis, and remodeling index. The age-and-BMI plus combined imaging model included age, body mass index, all four geometric parameters, and all four quantitative HR-VWI measurements. The reported AUCs represent apparent discrimination in the development cohort because no internal validation or calibration analysis was performed.

**FIGURE 1:**
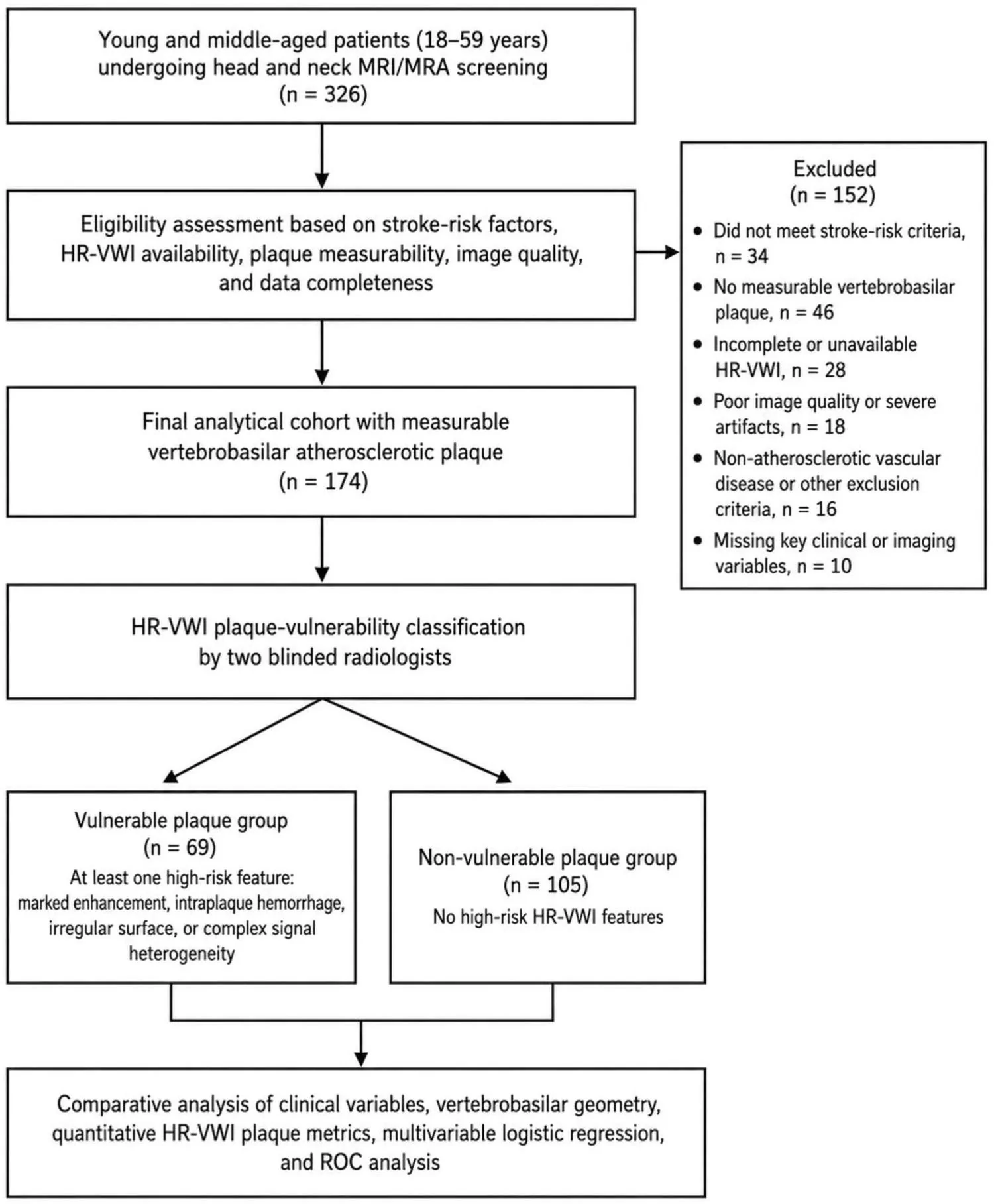
Cohort assembly and analytical workflow.

The study workflow is shown in Figure 1. The variable-selection procedure and final regression coefficients were not evaluated using bootstrap resampling. Therefore, selection stability, coefficient optimism, and optimism-corrected model performance were not estimated, and the final model was regarded as exploratory.

A sensitivity analysis was conducted to evaluate the potential influence of the outcome-dependent index-plaque selection strategy. For this analysis, one plaque was selected for each participant using a uniform rule: the measurable vertebrobasilar plaque with the greatest plaque burden was selected irrespective of qualitative vulnerability features or participant group assignment. When plaques had identical plaque burdens, the plaque with the greater wall area and then the greater percentage area stenosis was selected. The participant-level outcome remained the presence of at least one HR-VWI-defined vulnerable plaque. Quantitative HR-VWI measurements from the uniformly selected plaque were substituted for those used in the primary analysis, whereas the clinical and vertebrobasilar geometric variables remained unchanged. To isolate the effect of plaque selection from that of variable-selection instability, the sensitivity analysis used the fixed predictor specification from the primary final model without repeating backward elimination. The four prespecified classification models were also refitted using measurements from the uniformly selected plaques.

## 3 Results

### 3.1 Study cohort and baseline characteristics

Among 326 young and middle-aged individuals who underwent head and neck MRI/MRA screening, 174 patients with measurable vertebrobasilar atherosclerotic plaques and eligible high-resolution vessel wall imaging (HR-VWI) data were included in the final analysis. According to predefined HR-VWI plaque-vulnerability criteria, 69 patients were assigned to the vulnerable plaque group and 105 patients to the non-vulnerable plaque group. All 174 participants were inpatients. Dizziness or vertigo was the most frequent primary admission diagnosis, accounting for 52 patients (29.9%), followed by headache in 31 (17.8%), transient ischemic attack or transient focal neurological symptoms in 27 (15.5%), acute ischemic stroke in 24 (13.8%), and cerebral atherosclerosis or cerebrovascular-risk evaluation in 21 (12.1%). Other neurological and non-neurological disorders accounted for 12 (6.9%) and 7 (4.0%) admissions, respectively. The complete distribution is presented in Supplementary Table 3.

Baseline clinical characteristics are summarized in Table 1. Patients with vulnerable plaques were older than those with non-vulnerable plaques (48.4 ± 7.0 years vs. 45.9 ± 7.6 years; *P* = 0.032) and had a higher body mass index (25.9 ± 3.1 kg/m^2^ vs. 24.8 ± 3.0 kg/m^2^; *P* = 0.021). The corresponding standardized differences for age and body mass index were 0.34 and 0.36, respectively, indicating small-to-moderate between-group imbalance. The proportions of male sex, hypertension, diabetes mellitus, dyslipidemia, current smoking, alcohol consumption, and previous transient ischemic attack or ischemic stroke were numerically higher in the vulnerable plaque group, but these differences did not reach statistical significance.

### 3.2 Vertebrobasilar geometric characteristics associated with plaque vulnerability

**FIGURE 2:**
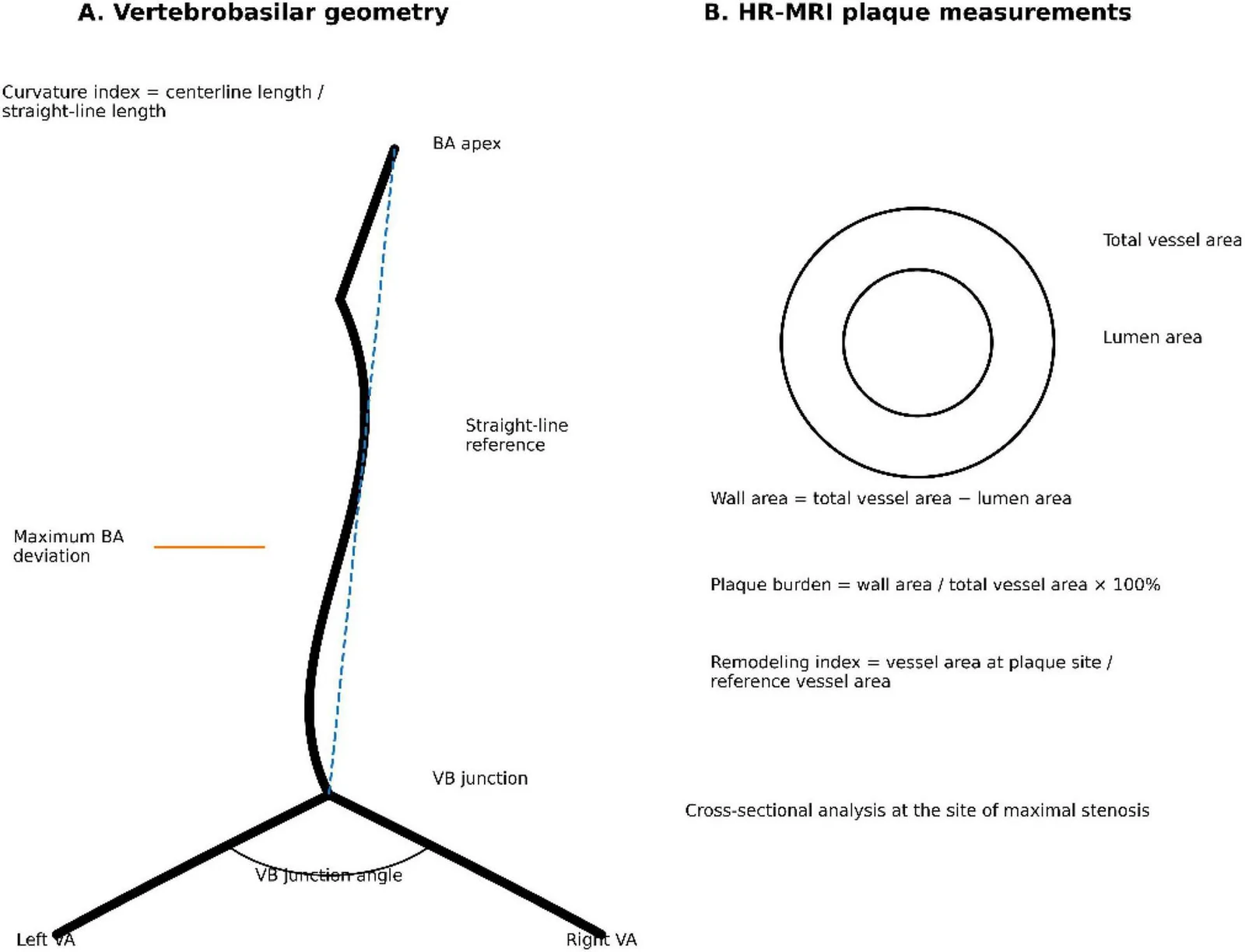
Quantification of vertebrobasilar arterial geometry and plaque morphology using high-resolution vessel-wall MRI. **(A)** Schematic representation of vertebrobasilar geometry, including the basilar artery curvature index, vertebrobasilar junction angle, bilateral V4-segment diameter difference, and maximum basilar artery deviation. The curvature index was calculated as the ratio of the BA centerline length to the straight-line distance between the VB junction and BA apex. Maximum BA deviation was defined as the greatest perpendicular distance between the BA centerline and the straight-line reference. **(B)** Cross-sectional HR-VWI plaque measurements, including lumen area, total vessel area, wall area, plaque burden, percentage area stenosis, and remodeling index. BA, basilar artery; HR-VWI, high-resolution vessel-wall imaging; V4, fourth or intracranial segment of the vertebral artery; VB, vertebrobasilar.

**FIGURE 3:**
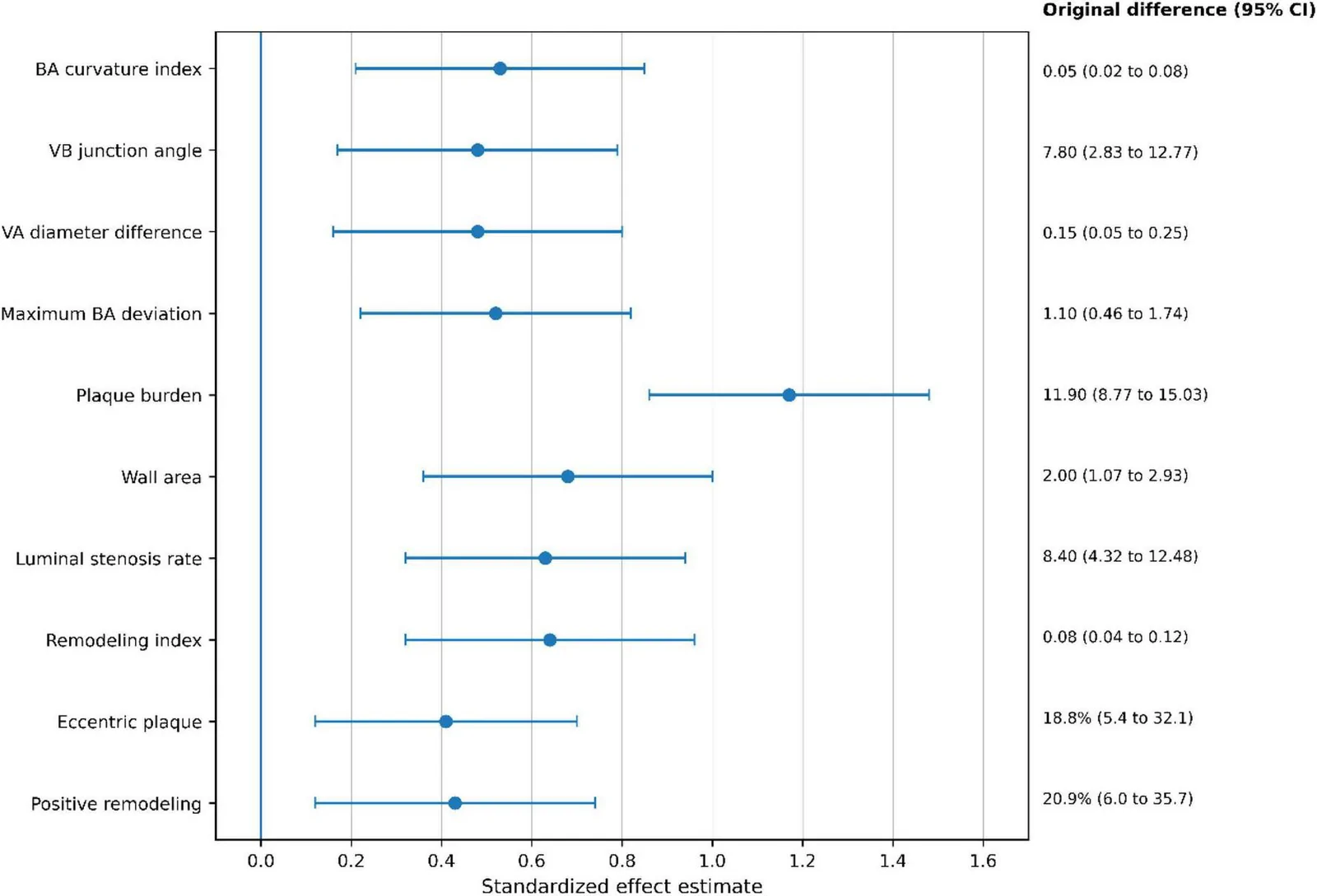
Effect-size profile of vertebrobasilar geometry and HR-VWI markers associated with plaque vulnerability.

The vertebrobasilar geometric and plaque-quantification framework is illustrated in Figure 2. Quantitative comparisons of vertebrobasilar geometry between the vulnerable and non-vulnerable plaque groups are presented in Table 2 and visualized as standardized effect estimates in Figure 3. Compared with the non-vulnerable plaque group, the vulnerable plaque group showed a significantly higher basilar artery curvature index (1.20 ± 0.10 vs. 1.15 ± 0.09; mean difference, 0.05; 95% CI, 0.02–0.08; *P* < 0.001), a larger vertebrobasilar junction angle (66.9 ± 16.8° vs. 59.1 ± 15.7°; mean difference, 7.80°; 95% CI, 2.83–12.77; *P* = 0.002), and a greater bilateral vertebral artery diameter difference (0.72 ± 0.33 mm vs. 0.57 ± 0.30 mm; mean difference, 0.15 mm; 95% CI, 0.05–0.25; *P* = 0.002). Maximum basilar artery deviation was also significantly greater in the vulnerable plaque group than in the non-vulnerable plaque group (5.4 ± 2.2 mm vs. 4.3 ± 2.0 mm; mean difference, 1.10 mm; 95% CI, 0.46–1.74; *P* < 0.001).

**TABLE 2:** Vertebrobasilar geometry and HR-MRI plaque characteristics according to plaque vulnerability.

Imaging variable	Vulnerable plaque group (*n* = 69)	Non-vulnerable plaque group (*n* = 105)	Difference (95% CI)	*P*-value
Vertebrobasilar geometric parameters				
Basilar artery curvature index	1.20 ± 0.10	1.15 ± 0.09	0.05 (0.02–0.08)	< 0.001
Vertebrobasilar junction angle, °	66.9 ± 16.8	59.1 ± 15.7	7.80 (2.83–12.77)	0.002
Basilar artery length, mm	32.0 ± 4.4	30.7 ± 4.2	1.30 (−0.01 to 2.61)	0.051
Mean basilar artery diameter, mm	3.15 ± 0.45	3.06 ± 0.42	0.09 (−0.04 to 0.22)	0.180
Bilateral vertebral artery diameter difference, mm	0.72 ± 0.33	0.57 ± 0.30	0.15 (0.05–0.25)	0.002
Maximum basilar artery deviation, mm	5.4 ± 2.2	4.3 ± 2.0	1.10 (0.46–1.74)	< 0.001
Quantitative HR- VWI plaque metrics				
Plaque burden, %	60.9 ± 10.7	49.0 ± 9.7	11.90 (8.77–15.03)	< 0.001
Wall area, mm^2^	13.1 ± 3.2	11.1 ± 2.8	2.00 (1.07–2.93)	< 0.001
Percentage area stenosis, %	39.8 ± 13.9	31.4 ± 12.7	8.40 (4.32–12.48)	< 0.001
Remodeling index	1.12 ± 0.13	1.04 ± 0.12	0.08 (0.04–0.12)	< 0.001
Lumen area, mm^2^	8.9 ± 2.5	9.7 ± 2.6	−0.80 (−1.57 to −0.03)	0.045
Total vessel area, mm^2^	22.0 ± 4.5	20.8 ± 4.2	1.20 (−0.13 to 2.53)	0.073
Qualitative plaque phenotype				
Eccentric plaque	55/69 (79.7)	64/105 (61.0)	18.8% (5.4–32.1)	0.009
Positive remodeling	42/69 (60.9)	42/105 (40.0)	20.9% (6.0–35.7)	0.007
Negative remodeling	9/69 (13.0)	25/105 (23.8)	−10.8% (−22.1 to 0.6)	0.080
Marked plaque enhancement	43/69 (62.3)	0/105 (0.0)	62.3% (50.9–73.8)	–
Irregular plaque surface	20/69 (29.0)	0/105 (0.0)	29.0% (18.3–39.7)	–
Intraplaque hemorrhage	13/69 (18.8)	0/105 (0.0)	18.8% (9.6–28.1)	–
Complex/mixed signal pattern	27/69 (39.1)	0/105 (0.0)	39.1% (27.6–50.6)	–

Continuous variables are presented as mean ± SD, with mean difference and 95% CI. Categorical variables are presented as n/N (%), with absolute percentage difference and 95% CI. HR-MRI, high-resolution magnetic resonance imaging; CI, confidence interval.

Basilar artery length showed a borderline difference between groups, with a longer mean length in the vulnerable plaque group (32.0 ± 4.4 mm vs. 30.7 ± 4.2 mm; mean difference, 1.30 mm; 95% CI, −0.01 to 2.61; *P* = 0.051). Mean basilar artery diameter did not differ significantly between groups (3.15 ± 0.45 mm vs. 3.06 ± 0.42 mm; mean difference, 0.09 mm; 95% CI, −0.04 to 0.22; *P* = 0.180). These findings indicate that plaque vulnerability was more closely related to curvature, confluence geometry, vertebral artery asymmetry, and basilar artery lateral deviation than to mean basilar artery caliber.

### 3.3 Quantitative HR-VWI plaque features according to vulnerability status

Quantitative HR-VWI plaque measurements differed substantially between vulnerable and non-vulnerable plaques (Table 2 and Figure 3). The vulnerable plaque group had a markedly higher plaque burden than the non-vulnerable plaque group (60.9 ± 10.7% vs. 49.0 ± 9.7%; mean difference, 11.90%; 95% CI, 8.77–15.03; *P* < 0.001). Wall area was also significantly larger in the vulnerable plaque group (13.1 ± 3.2 mm^2^ vs. 11.1 ± 2.8 mm^2^; mean difference, 2.00 mm^2^; 95% CI, 1.07–2.93; *P* < 0.001). In addition, vulnerable plaques were associated with a greater percentage area stenosis (39.8 ± 13.9% vs. 31.4 ± 12.7%; mean difference, 8.40%; 95% CI, 4.32–12.48; *P* < 0.001) and a higher remodeling index (1.12 ± 0.13 vs. 1.04 ± 0.12; mean difference, 0.08; 95% CI, 0.04–0.12; *P* < 0.001).

Lumen area was smaller in the vulnerable plaque group than in the non-vulnerable plaque group (8.9 ± 2.5 mm^2^ vs. 9.7 ± 2.6 mm^2^; mean difference, −0.80 mm^2^; 95% CI, −1.57 to −0.03; *P* = 0.045), whereas total vessel area showed no statistically significant difference (22.0 ± 4.5 mm^2^ vs. 20.8 ± 4.2 mm^2^; mean difference, 1.20 mm^2^; 95% CI, −0.13 to 2.53; *P* = 0.073). These results suggest that vulnerable plaques were characterized by greater wall disease burden and more severe luminal compromise, accompanied by a tendency toward outward remodeling.

### 3.4 Qualitative HR-VWI plaque phenotypes

**FIGURE 4:**
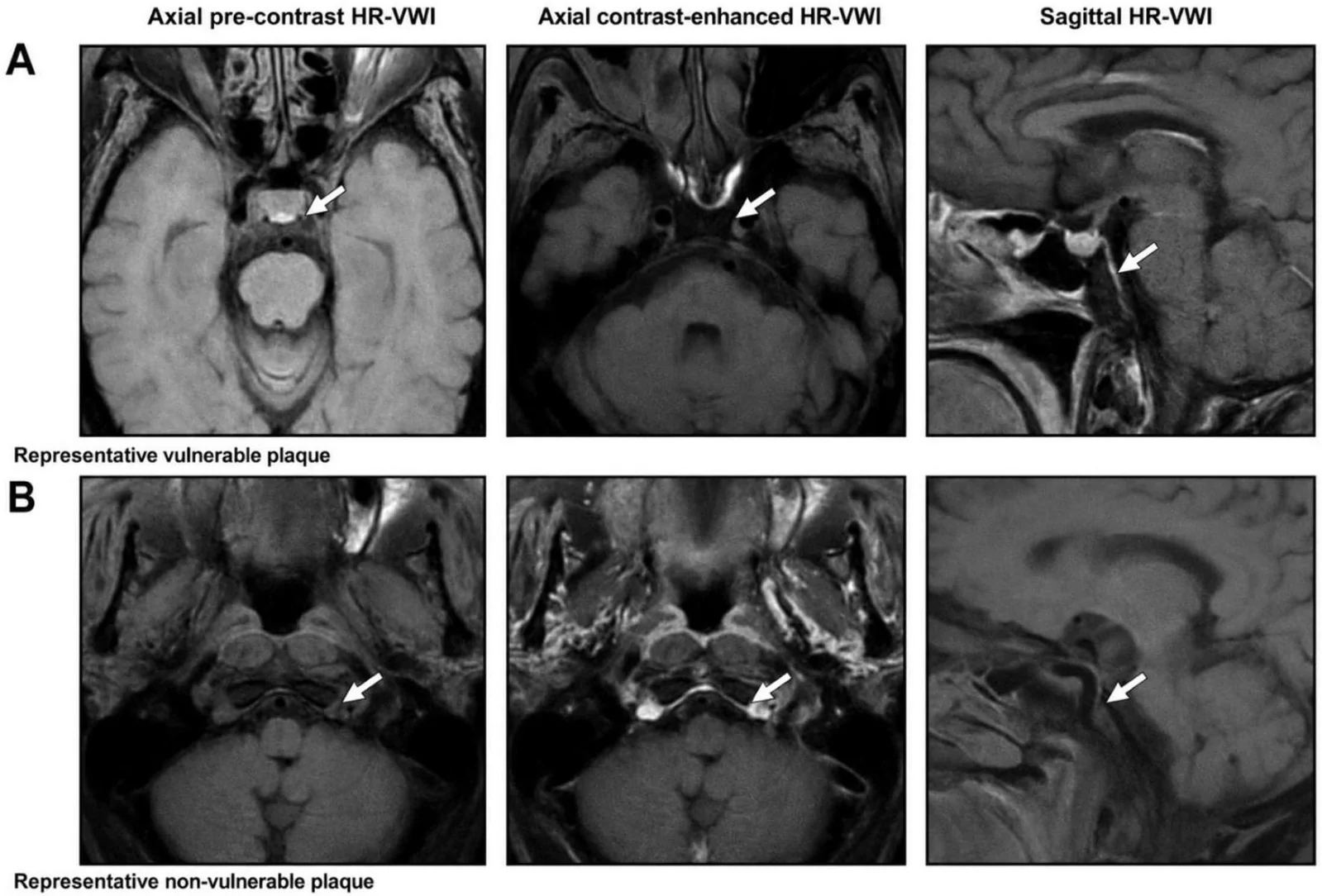
Vessel-wall MRI phenotypes of vulnerable and non-vulnerable vertebrobasilar plaques. **(A1)** Axial pre-contrast HR-VWI showing a representative vulnerable plaque. **(A2)** Axial contrast-enhanced HR-VWI showing plaque enhancement in the vulnerable plaque. **(A3)** Sagittal HR-VWI showing longitudinal localization of the vulnerable plaque. **(B1)** Axial pre-contrast HR-VWI showing a representative non-vulnerable plaque. **(B2)** Axial contrast-enhanced HR-VWI showing absent or mild enhancement in the non-vulnerable plaque. **(B3)** Sagittal HR-VWI showing longitudinal localization of the non-vulnerable plaque.

Representative HR-VWI examples of vulnerable and non-vulnerable vertebrobasilar plaques are shown in Figure 4. The vulnerable plaque example demonstrated conspicuous plaque enhancement on contrast-enhanced HR-VWI, whereas the non-vulnerable plaque showed absent or mild enhancement without additional high-risk vessel-wall features.

Qualitative plaque phenotypes are summarized in Table 2 and further represented in Figure 3. Eccentric plaques were more frequent in the vulnerable plaque group than in the non-vulnerable plaque group (79.7% vs. 61.0%; absolute difference, 18.8%; 95% CI, 5.4–32.1; *P* = 0.009). Positive remodeling was also more common in the vulnerable plaque group (60.9% vs. 40.0%; absolute difference, 20.9%; 95% CI, 6.0–35.7; *P* = 0.007), whereas negative remodeling was numerically less frequent but did not reach statistical significance (13.0% vs. 23.8%; absolute difference, −10.8%; 95% CI, −22.1 to 0.6; *P* = 0.080).

As expected from the prespecified vulnerability definition, marked plaque enhancement, irregular plaque surface, intraplaque hemorrhage, and complex or mixed signal pattern were observed only in the vulnerable plaque group. Marked enhancement was present in 62.3% of vulnerable plaques, irregular plaque surface in 29.0%, intraplaque hemorrhage in 18.8%, and complex or mixed signal pattern in 39.1%. These features were interpreted descriptively and were not entered into the multivariable prediction model to avoid circular prediction.

### 3.5 Anatomical site-based comparison of vertebrobasilar plaques

**FIGURE 5:**
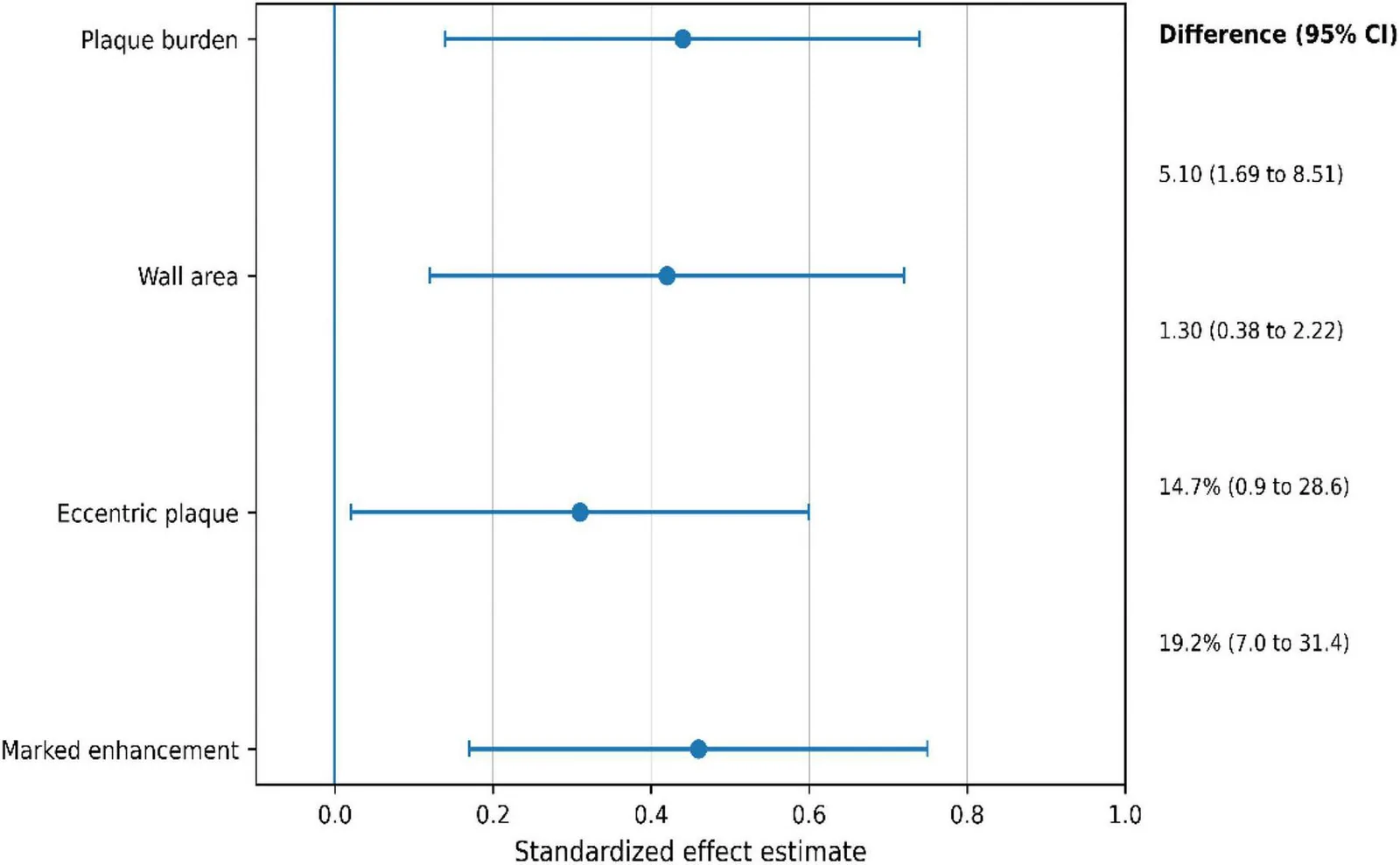
Location-dependent plaque phenotype in the vertebrobasilar system.

Anatomical site-based plaque comparisons are shown in Table 3 and Figure 5. Among the 174 index plaques analyzed, 96 were located in the basilar artery and 78 in the V4 segment of the vertebral artery. Basilar artery plaques showed higher plaque burden than V4-segment vertebral artery plaques (56.6 ± 11.8% vs. 51.5 ± 11.1%; mean difference, 5.10%; 95% CI, 1.69–8.51; *P* = 0.008). Wall area was also larger in basilar artery plaques (12.6 ± 2.9 mm^2^ vs. 11.3 ± 3.2 mm^2^; mean difference, 1.30 mm^2^; 95% CI, 0.38–2.22; *P* = 0.006).

**TABLE 3:** Anatomical site-based comparison of basilar artery and V4-segment vertebral artery plaques.

Plaque characteristic	Basilar artery plaques (*n* = 96)	V4-segment vertebral artery plaques (*n* = 78)	Difference (95% CI)	*P*-value
Plaque burden, %	56.6 ± 11.8	51.5 ± 11.1	5.10 (1.69–8.51)	0.008
Percentage area stenosis, %	36.6 ± 13.7	32.8 ± 13.7	3.80 (−0.29 to 7.89)	0.070
Wall area, mm^2^	12.6 ± 2.9	11.3 ± 3.2	1.30 (0.38–2.22)	0.006
Remodeling index	1.08 ± 0.14	1.05 ± 0.12	0.03 (−0.01 to 0.07)	0.078
Eccentric plaque	72/96 (75.0)	47/78 (60.3)	14.7% (0.9–28.6)	0.038
Vulnerable plaque	43/96 (44.8)	26/78 (33.3)	11.5% (−3.0 to 25.9)	0.124
Marked enhancement	32/96 (33.3)	11/78 (14.1)	19.2% (7.0–31.4)	0.013

Continuous variables are presented as mean ± SD, with mean difference and 95% CI. Categorical variables are presented as n/N (%), with absolute percentage difference and 95% CI.

Basilar artery plaques more frequently showed eccentric morphology than V4-segment plaques (75.0% vs. 60.3%; absolute difference, 14.7%; 95% CI, 0.9–28.6; *P* = 0.038). Marked enhancement was also more common in basilar artery plaques (33.3% vs. 14.1%; absolute difference, 19.2%; 95% CI, 7.0–31.4; *P* = 0.013). Percentage area stenosis, remodeling index, and the overall proportion of vulnerable plaques were numerically higher in basilar artery plaques, but these differences did not reach statistical significance. These findings indicate that plaque phenotype varies according to anatomical location within the vertebrobasilar system, with basilar artery plaques showing a greater wall burden and more frequent high-risk morphological features.

### 3.6 Age- and body mass index-adjusted factors associated with vulnerable plaque status

**FIGURE 6:**
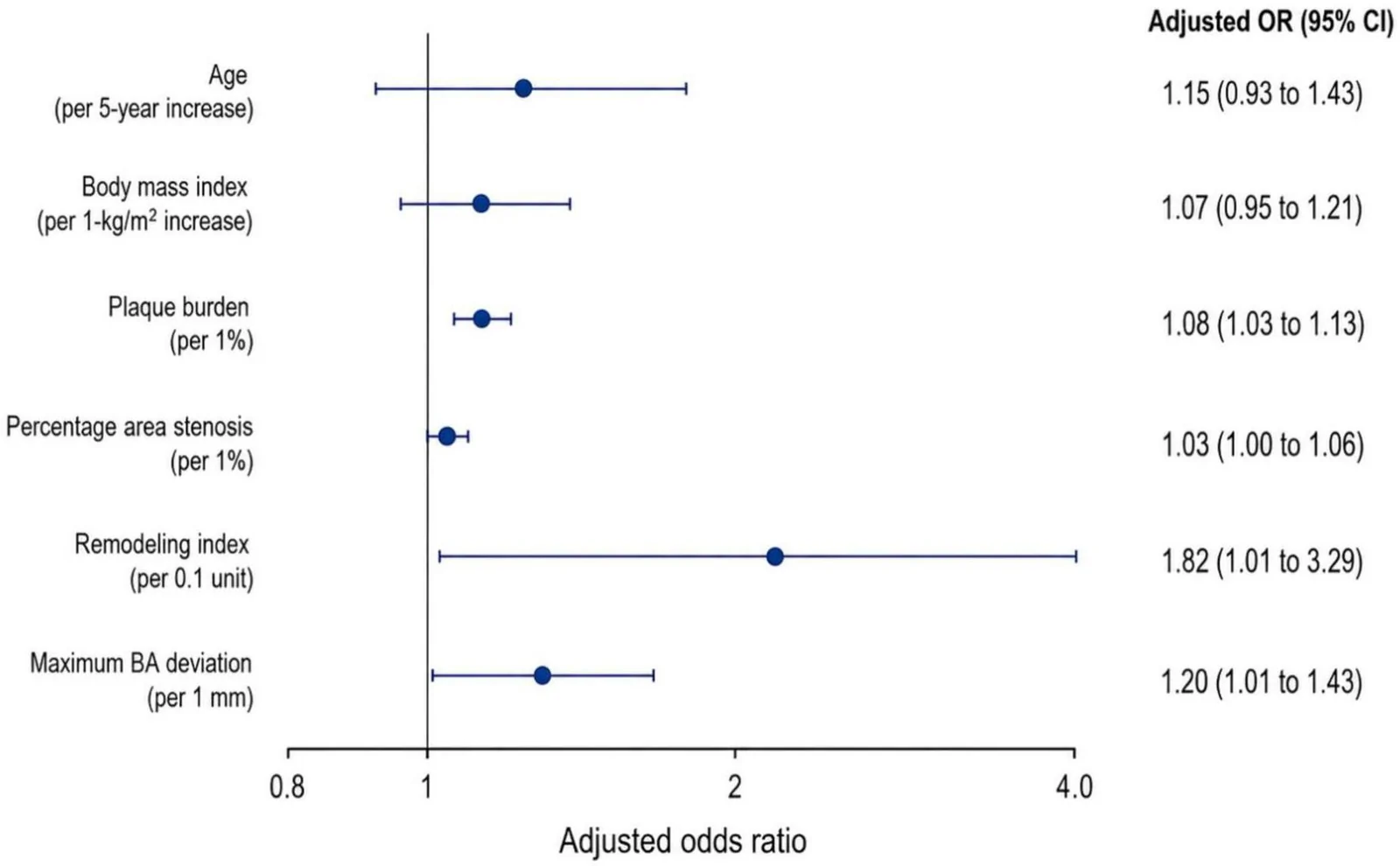
Independent imaging predictors of vulnerable plaque status.

The final multivariable logistic regression model is presented in Table 4 and visualized in the revised Figure 6. Age and body mass index were included as prespecified potential confounders and retained in the model irrespective of statistical significance. After simultaneous adjustment for these clinical covariates and the selected imaging variables, plaque burden, percentage area stenosis, remodeling index, and maximum basilar artery deviation remained associated with the HR-VWI-defined vulnerable plaque phenotype.

**TABLE 4:** Exploratory multivariable logistic regression analysis of factors associated with the HR-VWI-defined vulnerable plaque phenotype after adjustment for age and body mass index.

Predictor	Reporting unit	β	SE	Wald χ^2^	Adjusted OR (95% CI)	*P*-value
Age	Per 5-year increase	0.140	0.110	1.62	1.15 (0.93–1.43)	0.203
Body mass index	Per 1-kg/m^2^ increase	0.068	0.062	1.20	1.07 (0.95–1.21)	0.273
Plaque burden	Per 1% increase	0.077	0.024	10.60	1.08 (1.03–1.13)	0.001
Percentage area stenosis	Per 1% increase	0.030	0.015	3.95	1.03 (1.00–1.06)	0.047
Remodeling index	Per 0.1-unit increase	0.599	0.301	3.95	1.82 (1.01–3.29)	0.047
Maximum basilar artery deviation	Per 1-mm increase	0.182	0.089	4.22	1.20 (1.01–1.43)	0.040

The final model was obtained after univariable screening, collinearity assessment, and backward likelihood-ratio elimination. Age and body mass index were retained as clinically relevant covariates irrespective of statistical significance. Age was scaled per 5-year increase, and remodeling index was scaled per 0.1-unit increase to improve interpretability. The dependent variable was the presence of an HR-VWI-defined vulnerable plaque phenotype. β, regression coefficient; CI, confidence interval; HR-VWI, high-resolution vessel-wall imaging; OR, odds ratio; SE, standard error. Because the predictor-selection process was conducted in the development cohort without bootstrap validation, the selected variables and reported coefficients should be interpreted as exploratory.

Each 1% increase in plaque burden was associated with an 8% increase in the odds of vulnerable plaque status (adjusted OR, 1.08; 95% CI, 1.03–1.13; *P* = 0.001). Each 1% increase in percentage area stenosis was associated with an adjusted OR of 1.03 (95% CI, 1.00–1.06; *P* = 0.047). A 0.1-unit increase in remodeling index was associated with an adjusted OR of 1.82 (95% CI, 1.01–3.29; *P* = 0.047), whereas each 1-mm increase in maximum basilar artery deviation was associated with an adjusted OR of 1.20 (95% CI, 1.01–1.43; *P* = 0.040).

Age was not independently associated with vulnerable plaque status after adjustment for the imaging variables (adjusted OR per 5-year increase, 1.15; 95% CI, 0.93–1.43; *P* = 0.203). Body mass index was also not independently associated with the outcome (adjusted OR per 1-kg/m^2^ increase, 1.07; 95% CI, 0.95–1.21; *P* = 0.273). These findings indicate that the principal imaging associations persisted after accounting for the observed baseline differences in age and body mass index.

Univariable screening and collinearity diagnostics are summarized in Supplementary Table 5. Of the 14 prespecified candidate predictors, 13 met the univariable screening criterion of *P* < 0.10 or were retained as forced clinical covariates. Mean basilar artery diameter did not meet the screening threshold and was not entered into the multivariable analysis. Wall area, lumen area, and total vessel area showed potentially important collinearity, with VIF values greater than 5, and were excluded before backward elimination. Basilar artery length, basilar artery curvature index, vertebrobasilar junction angle, and bilateral vertebral artery diameter difference were subsequently removed during backward likelihood-ratio elimination. The final model retained age, body mass index, plaque burden, percentage area stenosis, remodeling index, and maximum basilar artery deviation. Final-model VIF values ranged from 1.09 to 2.08, indicating no important residual multicollinearity. Because the final predictor set was obtained through data-dependent selection without resampling, its selection stability and coefficient optimism could not be assessed.

### 3.7 Discrimination and pairwise DeLong comparisons of the clinical-adjusted models

**FIGURE 7:**
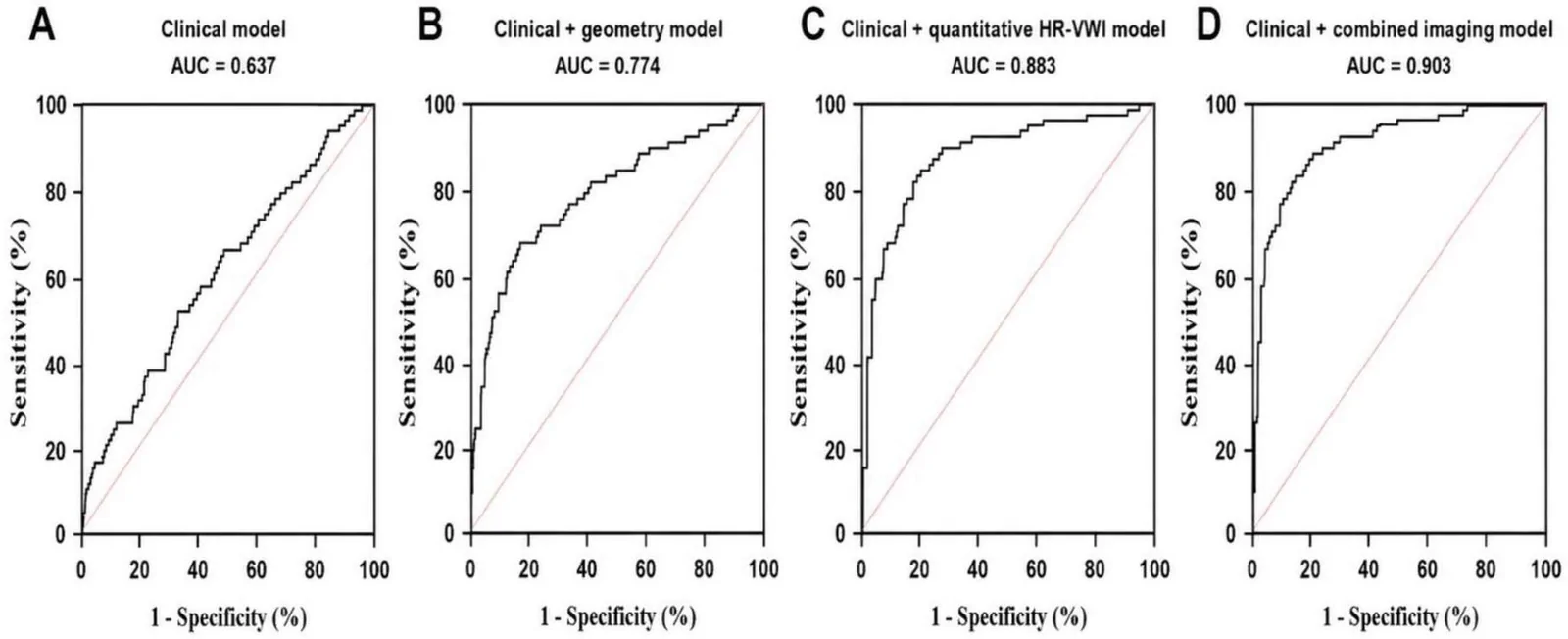
Receiver operating characteristic curves of the clinical-adjusted models for classifying the HR-VWI-defined vulnerable plaque phenotype. **(A)** Clinical model including age and body mass index; **(B)** clinical plus geometry model; **(C)** clinical plus quantitative HR-VWI model; and **(D)** clinical plus combined imaging model. The corresponding AUCs were 0.637, 0.774, 0.883, and 0.903. DeLong testing showed significant differences between each imaging-augmented model and the clinical model (*P* = 0.003, *P* < 0.001, and *P* < 0.001, respectively). The difference between the clinical plus combined imaging model and the clinical plus quantitative HR-VWI model was not statistically significant (ΔAUC = 0.020; *P* = 0.162). The diagonal line indicates chance-level discrimination. AUC, area under the receiver operating characteristic curve; HR-VWI, high-resolution vessel-wall imaging.

The discriminative performance of the four clinical-adjusted classification models is summarized in Table 5A and Figure 7, while the prespecified pairwise DeLong comparisons are presented in Table 5B. These models classified the contemporaneously assessed HR-VWI-defined vulnerable plaque phenotype and should not be interpreted as predicting future ischemic events.

**TABLE 5A:** Apparent development-cohort discrimination of the age-and-BMI-adjusted models for classifying the HR-VWI-defined vulnerable plaque phenotype.

Model	Included predictor domains	AUC (95% CI)	Optimal probability cutoff	Sensitivity, %	Specificity, %	Youden index	ΔAUC versus clinical model	*P*-value
Clinical model	Age and body mass index	0.637 (0.554–0.720)	> 0.387	62.32	62.86	0.252	Reference	–
Clinical + geometry model	Age, body mass index, basilar artery curvature index, vertebrobasilar junction angle, vertebral artery diameter difference, and maximum basilar artery deviation	0.774 (0.705–0.843)	> 0.426	69.57	80.95	0.505	0.137	0.003
Clinical + quantitative HR-VWI model	Age, body mass index, plaque burden, wall area, percentage area stenosis, and remodeling index	0.883 (0.832–0.934)	> 0.351	86.96	76.19	0.632	0.246	< 0.001
Clinical + combined imaging model	Age, body mass index, basilar artery curvature index, vertebrobasilar junction angle, bilateral vertebral artery diameter difference, maximum basilar artery deviation, plaque burden, wall area, percentage area stenosis, and remodeling index	0.903 (0.858–0.948)	> 0.506	78.26	89.52	0.678	0.266	< 0.001

Values represent apparent discrimination estimates obtained from the development cohort; internal validation and calibration were not performed. Optimal probability thresholds were determined by maximizing the Youden index. The reported sensitivity and specificity correspond to the indicated thresholds. *P*-values represent DeLong comparisons with the age-and-BMI model. The age-and-BMI plus combined imaging model was also compared with the age-and-BMI plus quantitative HR-VWI model (ΔAUC = 0.020; *P* = 0.162).

**TABLE 5B:** Pairwise comparisons of correlated AUCs using the DeLong test.

Model comparison	AUC of first model	AUC of second model	Absolute ΔAUC	DeLong *p*-value
Clinical plus geometry vs. clinical	0.774	0.637	0.137	0.003
Clinical plus quantitative HR-VWI vs. clinical	0.883	0.637	0.246	< 0.001
Clinical plus combined imaging vs. clinical	0.903	0.637	0.266	< 0.001
Clinical plus combined imaging vs. clinical plus quantitative HR-VWI	0.903	0.883	0.020	0.162

AUCs were compared using the nonparametric DeLong test for correlated ROC curves because all models were evaluated in the same participants. Comparisons with the clinical model assessed the incremental discriminatory contribution of each imaging domain. The comparison between the clinical plus combined imaging model and the clinical plus quantitative HR-VWI model evaluated whether vertebrobasilar geometry provided additional discrimination beyond quantitative HR-VWI measurements. AUC, area under the receiver operating characteristic curve; CI, confidence interval; HR-VWI, high-resolution vessel-wall imaging; ROC, receiver operating characteristic.

The clinical model containing age and body mass index showed limited discrimination, with an AUC of 0.637 (95% CI, 0.554–0.720). The clinical plus geometry model demonstrated an AUC of 0.774 (95% CI, 0.705–0.843), representing an absolute improvement of 0.137 over the clinical model. This difference was statistically significant according to the DeLong test (*P* = 0.003).

The clinical plus quantitative HR-VWI model had an AUC of 0.883 (95% CI, 0.832–0.934), representing an absolute increase of 0.246 compared with the clinical model (DeLong *P* < 0.001). The clinical plus combined imaging model showed the highest numerical AUC of 0.903 (95% CI, 0.858–0.948), which was also significantly higher than that of the clinical model (ΔAUC = 0.266; DeLong *P* < 0.001).

However, the AUC difference between the clinical plus combined imaging model and the clinical plus quantitative HR-VWI model was only 0.020 and was not statistically significant according to the DeLong test (*P* = 0.162). Therefore, although the combined model had the highest numerical AUC, the analysis did not demonstrate a statistically significant incremental improvement in overall discrimination from adding vertebrobasilar geometry to the clinical plus quantitative HR-VWI model.

At their respective optimal thresholds, the combined model had higher specificity than the clinical plus quantitative HR-VWI model (89.52% vs. 76.19%) but lower sensitivity (78.26% vs. 86.96%). Thus, the addition of geometric parameters primarily altered the sensitivity–specificity balance rather than significantly increasing the AUC.

### 3.8 Interobserver reproducibility

Interobserver reproducibility results are summarized in Supplementary Table 4. Quantitative vertebrobasilar geometric and plaque measurements demonstrated good-to-excellent agreement, with ICCs ranging from 0.877 for percentage area stenosis to 0.948 for basilar artery length. The ICCs for the basilar artery curvature index, vertebrobasilar junction angle, mean basilar artery diameter, and wall area were greater than 0.90, indicating excellent reproducibility. The remaining quantitative parameters showed good agreement, with ICCs ranging from 0.877 to 0.898.

Agreement for qualitative plaque features was almost perfect, with kappa coefficients ranging from 0.834 for intraplaque hemorrhage to 0.947 for basilar artery bending grade. Overall percentage agreement ranged from 90.8 to 97.7%. Agreement for the final HR-VWI-defined vulnerable plaque phenotype was also almost perfect, with an overall agreement of 95.4% and a kappa coefficient of 0.904 (95% CI, 0.839–0.961). These findings indicate high interobserver reliability for the principal geometric, quantitative, and qualitative imaging assessments.

### 3.9 Sensitivity analysis using a uniform index-plaque selection rule

Forty-eight of the 174 participants (27.6%) had more than one measurable vertebrobasilar plaque, including 29 participants with and 19 participants without the HR-VWI-defined vulnerable plaque phenotype. Application of the uniform largest-plaque rule changed the selected index plaque in 13 participants (7.5% of the total cohort), all of whom belonged to the vulnerable-plaque group. The selected plaque remained unchanged in the other 161 participants (92.5%).

After application of the uniform selection rule, participants with the vulnerable plaque phenotype continued to demonstrate greater plaque burden, wall area, percentage area stenosis, and remodeling index than participants without the vulnerable phenotype. The between-group differences were slightly attenuated compared with the primary analysis but remained statistically significant.

In the fixed multivariable sensitivity model, the directions and magnitudes of the principal associations were broadly consistent with those of the primary analysis. Plaque burden remained associated with the vulnerable plaque phenotype (adjusted OR per 1% increase, 1.07; 95% CI, 1.03–1.12; *P* = 0.002), as did percentage area stenosis (adjusted OR per 1% increase, 1.03; 95% CI, 1.00–1.06; *P* = 0.041), remodeling index (adjusted OR per 0.1-unit increase, 1.76; 95% CI, 1.02–3.04; *P* = 0.043), and maximum basilar artery deviation (adjusted OR per 1-mm increase, 1.18; 95% CI, 1.01–1.39; *P* = 0.037). Age and body mass index were not statistically significant.

The age-and-BMI plus quantitative HR-VWI model had an AUC of 0.872 (95% CI, 0.817–0.927), while the age-and-BMI plus combined imaging model had an AUC of 0.894 (95% CI, 0.846–0.942). The AUC difference between these two models remained nonsignificant (ΔAUC = 0.022; DeLong *P* = 0.148). Thus, use of a uniform plaque-selection rule produced modest attenuation of the quantitative estimates but did not materially alter the direction of the associations or the comparative discrimination of the imaging models.

## 4 Discussion

This study examined the relationship between vertebrobasilar geometry, quantitative HR-VWI measurements, and an HR-VWI-defined vulnerable plaque phenotype in young and middle-aged adults with stroke risk factors. Four principal findings emerged. First, vulnerable plaques were associated with greater basilar artery curvature, a larger vertebrobasilar junction angle, greater vertebral artery diameter asymmetry, and increased maximum basilar artery deviation. Second, vulnerable plaques had greater plaque burden, wall area, percentage area stenosis, and remodeling index. Third, plaque phenotype differed by anatomical location. Basilar artery plaques showed greater wall burden and more frequent adverse morphological features than V4-segment plaques. Finally, the clinical plus combined imaging model had the highest numerical AUC. However, its discrimination was not significantly greater than that of the clinical plus quantitative HR-VWI model.

### 4.1 Vertebrobasilar geometry and plaque vulnerability

Vulnerable plaques were associated with a more adverse vertebrobasilar geometric profile. The vulnerable plaque group had a higher basilar artery curvature index, larger vertebrobasilar junction angle, greater vertebral artery diameter difference, and greater maximum basilar artery deviation.

These findings may reflect disturbed posterior-circulation hemodynamics. Curvature and asymmetric vertebral inflow can promote secondary flow, flow separation, and spatial variation in wall shear stress. Such disturbances may contribute to endothelial dysfunction, inflammation, lipid deposition, and extracellular-matrix remodeling.

Previous studies have also linked vertebrobasilar configuration with plaque formation and posterior-circulation ischemia (9, 24–27). Xiong et al. reported associations between vertebrobasilar morphology, basilar artery position, and high-risk plaque characteristics (9). Other studies have shown that arterial curvature and lateral displacement are related to plaque distribution and posterior-circulation ischemic abnormalities (24–27). The present findings extend this evidence by demonstrating an association between maximum basilar artery deviation and the study-defined vulnerable plaque phenotype after adjustment for age and body mass index.

Vertebral artery diameter asymmetry may also have a mechanistic role. Unequal inflow from the two vertebral arteries can produce eccentric flow impingement at the vertebrobasilar junction. This may generate non-uniform wall shear stress along the basilar artery. The resulting hemodynamic environment could favor eccentric plaque formation and localized vessel-wall remodeling. These interpretations remain mechanistic hypotheses because flow parameters were not measured directly.

### 4.2 Quantitative HR-VWI characteristics

Quantitative HR-VWI measurements differed substantially between the two plaque groups. Vulnerable plaques had greater plaque burden, wall area, percentage area stenosis, and remodeling index. They also had a smaller lumen area.

Greater plaque burden and wall area indicate more extensive vessel-wall disease. A higher remodeling index suggests outward or positive remodeling. This process may preserve the lumen during plaque growth, but it can also accompany biologically active plaque expansion (28–30).

Previous HR-VWI studies have associated plaque burden, enhancement, intraplaque hemorrhage, surface irregularity, and positive remodeling with symptomatic intracranial atherosclerosis (13–16, 24, 25, 31). In the present study, plaque burden, percentage area stenosis, and remodeling index remained associated with vulnerable plaque status after adjustment for age and body mass index. The qualitative features used to define vulnerability were excluded from the model. This avoided direct incorporation of outcome-defining characteristics into the predictor set.

Luminal narrowing remained relevant but did not fully characterize plaque phenotype. Positive remodeling can preserve lumen caliber despite substantial wall disease. Therefore, lumen-based imaging alone may underestimate atherosclerotic burden. HR-VWI provides additional information by directly depicting the arterial wall.

### 4.3 Qualitative plaque features and biological interpretation

Eccentric morphology and positive remodeling were more frequent among vulnerable plaques. These characteristics may reflect focal and asymmetric mechanical loading of the vessel wall. In curved or laterally displaced arteries, disturbed flow may promote localized wall thickening and eccentric plaque growth.

Marked enhancement, intraplaque hemorrhage, irregular surface morphology, and complex signal heterogeneity were components of the vulnerability definition. Their concentration in the vulnerable group was therefore expected. These findings should be interpreted descriptively rather than as independent validation of the classification.

Plaque enhancement may reflect inflammation, endothelial permeability, neovascularity, or contrast leakage. Intraplaque hemorrhage and surface irregularity may indicate complex plaque composition or structural instability. However, histopathological confirmation was unavailable. The term “vulnerable plaque” therefore refers to the prespecified HR-VWI phenotype rather than pathologically proven instability.

Abnormal geometry and adverse plaque phenotype may be linked through a mechanobiological pathway. Disturbed flow may activate endothelial inflammatory signaling and increase vascular permeability. These changes may promote lipid accumulation, macrophage recruitment, and matrix degradation. Direct computational and biological evaluation is needed to confirm this proposed pathway.

### 4.4 Anatomical heterogeneity between basilar artery and V4 plaques

Plaque characteristics differed according to anatomical location. Basilar artery plaques had greater plaque burden and wall area than V4-segment plaques. They also showed more frequent eccentric morphology and marked enhancement.

Several anatomical and hemodynamic factors may explain these differences. The basilar artery receives converging flow from the two vertebral arteries. It is also influenced by local curvature, branch anatomy, perforator distribution, and vertebrobasilar junction geometry. These factors may create a heterogeneous flow environment that favors eccentric plaque growth and vessel-wall enhancement.

Previous studies have linked basilar artery geometry with plaque location and posterior-circulation infarction (28, 32–35). The present results support the importance of considering plaque location during HR-VWI interpretation. Nevertheless, the study was cross-sectional and cannot establish that anatomical location causes a more adverse plaque phenotype.

### 4.5 Interpretation of the clinical-adjusted models

The clinical model containing age and body mass index showed limited discrimination, with an AUC of 0.637. Addition of vertebrobasilar geometry increased the AUC to 0.774. Addition of quantitative HR-VWI measurements increased it to 0.883. DeLong testing showed that both imaging-augmented models performed significantly better than the clinical model.

The clinical plus combined imaging model had the highest numerical AUC of 0.903. However, the absolute increase over the clinical plus quantitative HR-VWI model was only 0.020. This difference was not statistically significant according to the DeLong test.

The combined model had higher specificity but lower sensitivity than the clinical plus quantitative HR-VWI model. Thus, adding geometric variables changed the sensitivity–specificity balance but did not significantly improve overall discrimination. This trade-off may be relevant for different imaging applications, but its clinical importance remains uncertain.

The reported AUCs should be interpreted as measures of within-examination discrimination of a prespecified HR-VWI phenotype. Although the quantitative imaging predictors were not components of the vulnerability definition, the imaging predictors and the outcome were derived from the same index imaging examination and were therefore not fully independent. This shared imaging source creates intrinsic biological and measurement dependence and may partly contribute to the observed discrimination. Accordingly, the models primarily evaluate concordance between quantitative imaging characteristics and a qualitative HR-VWI-defined phenotype—that is, internal imaging consistency rather than prediction of an independent histopathological, biological, or clinical endpoint. They should therefore be regarded as exploratory quantitative adjuncts and not as substitutes for direct expert interpretation or as models for predicting future ischemic events. Concurrent DWI-positive posterior-circulation infarction could, in principle, provide a clinically relevant external referent for the HR-VWI-defined plaque phenotype. However, the availability of DWI and ADC images alone is insufficient for reliable culprit-plaque classification. Such an analysis would require prespecified and blinded adjudication of infarct acuity and location, temporal correspondence with presenting symptoms, and anatomical concordance between the ischemic territory and a specific vertebral or basilar artery plaque. These elements were not systematically collected or adjudicated for the present study. The primary admission diagnosis could not serve as an equivalent substitute because an acute ischemic stroke diagnosis did not necessarily establish a DWI-positive posterior-circulation lesion attributable to the analyzed plaque. We therefore did not perform a *post hoc* symptomatic-versus-asymptomatic or DWI-referent analysis, which could otherwise introduce substantial outcome misclassification.

The sensitivity analysis provided additional information regarding the influence of the index-plaque selection strategy. When the measurable plaque with the greatest plaque burden was selected uniformly for every participant, irrespective of qualitative vulnerability status, the adjusted associations for plaque burden, percentage area stenosis, remodeling index, and maximum basilar artery deviation remained directionally consistent and of similar magnitude. The quantitative and combined imaging AUCs were modestly lower than those in the primary analysis, but the combined model continued to show no statistically significant improvement over the quantitative HR-VWI model. These findings suggest that the primary associations were not solely attributable to the outcome-dependent plaque-selection strategy, although the possibility of residual selection effects cannot be excluded.

### 4.6 Clinical and research implications

The findings have several implications for imaging research. First, quantitative HR-VWI measurements provide information beyond age and body mass index. Second, vertebrobasilar geometry may help characterize the vascular environment associated with plaque phenotype. Third, plaque location should be considered because basilar artery and V4-segment plaques showed different imaging characteristics.

However, the study does not establish a clinical decision rule. It did not evaluate treatment selection, management changes, net benefit, or longitudinal clinical outcomes. The applicability of these findings is also conditioned by the study population. Because all participants were inpatients selected after undergoing HR-VWI and demonstrating a measurable vertebrobasilar plaque, the results should be interpreted as characterizing an imaging-enriched clinical cohort rather than the full spectrum of intracranial atherosclerosis encountered in routine outpatient or community practice. Prospective studies should determine whether the identified imaging characteristics are associated with plaque progression or subsequent posterior-circulation ischemic events.

### 4.7 Limitations

This study has several limitations. First, this was a single-center study of a highly selected inpatient population rather than a population-based or routine-practice cohort of patients with intracranial atherosclerosis. Eligibility required referral for HR-VWI, the presence of at least one measurable nonocclusive vertebrobasilar plaque, sufficient image quality for delineation of the lumen and outer vessel-wall boundaries, and complete clinical and imaging data. This selection process may have preferentially included patients with symptoms, vascular risk factors, or imaging findings that prompted advanced vessel-wall imaging, while excluding outpatients, individuals with incidentally detected or less readily measurable disease, patients with complete occlusion, and those unable to undergo or complete diagnostic-quality MRI. Consequently, the prevalence and spectrum of plaque phenotypes, geometric characteristics, and quantitative HR-VWI measurements in this cohort may differ from those encountered in broader clinical practice. Such spectrum and referral selection may also influence the estimated associations and apparent model discrimination. Therefore, the findings are most directly applicable to similar inpatient populations undergoing technically adequate HR-VWI for measurable vertebrobasilar plaques and should not be generalized to all patients with intracranial atherosclerosis without external validation in multicenter, outpatient, community-based, and more clinically heterogeneous cohorts.

Second, the outcome was an HR-VWI-defined plaque phenotype rather than an independently established histopathological, biological, or clinical endpoint. Moreover, although the qualitative features defining the outcome were excluded from the predictor set, the imaging predictors and the outcome were obtained from the same index examination. Excluding the outcome-defining qualitative features avoided their direct incorporation into the models, but it did not eliminate the intrinsic dependence created by the shared imaging source. Consequently, the reported AUCs quantify contemporaneous within-examination classification, or internal imaging consistency, rather than external prediction. Their performance should not be interpreted as demonstrating an ability to predict pathologically confirmed plaque instability, plaque progression, treatment response, prognosis, or subsequent ischemic events. Qualitative HR-VWI features also permit direct identification of the study-defined phenotype; therefore, the models do not demonstrate superiority over expert image interpretation. Future longitudinal studies should evaluate these imaging characteristics against independent clinical outcomes or other external reference standards.

Third, the study lacked an independently adjudicated concurrent clinical or imaging referent. Although DWI and ADC sequences were included in the routine MRI protocol, DWI-positive posterior-circulation infarction and symptom–plaque territorial concordance were not prespecified or systematically adjudicated. Consequently, the study could not determine whether the HR-VWI-defined vulnerable phenotype or the model-generated probabilities were associated with a concurrent ischemic lesion in the corresponding vascular territory. This limits the clinical and biological interpretability of the term “vulnerable” and further supports describing the outcome specifically as an HR-VWI-defined plaque phenotype. Future studies should incorporate blinded assessment of DWI-positive posterior-circulation infarction, standardized culprit-plaque attribution, and symptomatic-versus-asymptomatic territorial classification as independent external outcomes.

Fourth, the multivariable association model was derived using univariable screening, collinearity-based exclusion, and backward likelihood-ratio selection in a cohort containing only 69 participants with the HR-VWI-defined vulnerable plaque phenotype. Although age and body mass index were prespecified and retained as forced covariates, selection of the remaining variables was data-dependent. With a modest number of outcome-positive participants relative to the number of candidate predictors considered during model development, the inclusion or exclusion of individual variables may be sensitive to small changes in the sample. Stepwise selection may also produce unstable regression coefficients, overly precise confidence intervals, and optimistic estimates of apparent discrimination. Accordingly, the selected predictor set and the magnitudes of the adjusted odds ratios should be interpreted as exploratory rather than as a definitive or reproducible set of independent predictors. Variables removed during selection should likewise not be interpreted as having no association with the imaging phenotype. Because the entire model-building process was not evaluated using bootstrap resampling, neither selection stability nor optimism-corrected performance was quantified. Future studies should prespecify parsimonious predictor sets, consider penalized or shrinkage-based regression methods, internally validate the complete model-development process using bootstrap resampling, assess calibration, and perform external validation in an independent cohort.

Fifth, HR-VWI measurements may be affected by spatial resolution, partial-volume effects, flow artifacts, vessel orientation, and manual boundary delineation. Although interobserver agreement was good to excellent, reproducibility across centers, scanners, and readers with different experience remains unknown.

Sixth, vertebrobasilar hemodynamics were inferred from anatomical geometry. Wall shear stress, oscillatory shear index, pressure gradients, and flow residence time were not measured directly.

Seventh, the primary index-plaque selection strategy differed according to vulnerability status and could therefore have preferentially selected more adverse plaque characteristics in the vulnerable group. To assess this concern, we performed a sensitivity analysis in which the measurable plaque with the greatest plaque burden was selected uniformly for all participants, irrespective of vulnerability status. The directions and magnitudes of the principal adjusted associations remained broadly consistent, and the comparative model discrimination was materially unchanged, although the quantitative and combined-model AUCs were modestly attenuated. These results reduce, but do not eliminate, concern regarding outcome-dependent selection bias. Moreover, only one plaque was analyzed per participant. An all-plaque lesion-level analysis would require systematic measurements of every plaque and clustered statistical methods to account for within-participant correlation.

Finally, adjustment was limited to age and body mass index. Residual confounding by other clinical, metabolic, treatment-related, and lifestyle factors cannot be excluded.

## 5 Conclusion

Vulnerable vertebrobasilar plaques in young and middle-aged individuals with stroke risk factors exhibited adverse geometric and vessel-wall imaging characteristics. In the exploratory selected multivariable model, plaque burden, percentage area stenosis, remodeling index, and maximum basilar artery deviation were retained and remained associated with the HR-VWI-defined vulnerable plaque phenotype after adjustment for age and body mass index. Because variable selection was performed in a modest development cohort without resampling, the stability of this predictor set requires confirmation in independent datasets. The clinical plus combined imaging model showed the highest numerical AUC; however, DeLong testing demonstrated that its discrimination was not significantly greater than that of the clinical plus quantitative HR-VWI model. Thus, the present results do not establish a statistically significant incremental benefit from adding vertebrobasilar geometry beyond clinical covariates and quantitative HR-VWI measurements. The geometric parameters may nevertheless influence the balance between sensitivity and specificity, a finding that requires validation using prospective outcomes and clinically relevant decision thresholds. More broadly, the reported discrimination reflects within-examination concordance between quantitative imaging characteristics and an HR-VWI-defined phenotype, rather than external prediction of histopathological plaque instability or future clinical events.

## Data Availability

The original contributions presented in the study are included in the article/Supplementary material, further inquiries can be directed to the corresponding author.

## References

[1] Gbd 2021 Stroke Risk Factor Collaborators. Global, regional, and national burden of stroke and its risk factors, 1990-2021: a systematic analysis for the global burden of disease study 2021. *Lancet Neurol.* (2024) 23:973–1003. 10.1016/S1474-4422(24)00369-7 39304265 PMC12254192

[2] Bártlová S Šedová L Havierniková L Hudáčková A Dolák F Sadílek P. Quality of life of post-stroke patients. *Zdr Varst.* (2022) 61:101–8. 10.2478/sjph-2022-0014 35432611 PMC8937589

[3] Bukhari S Yaghi S Bashir Z. Stroke in young adults. *J Clin Med.* (2023) 12:4999. 10.3390/jcm12154999 37568401 PMC10420127

[4] Boot E Ekker MS Putaala J Kittner S De Leeuw FE Tuladhar AM. Ischaemic stroke in young adults: a global perspective. *J Neurol Neurosurg Psychiatry.* (2020) 91:411–7. 10.1136/jnnp-2019-322424 32015089

[5] Yu Q Tian Y Jiang N Zhao F Wang S Sun M et al. Global, regional, and national burden and trends of stroke among youths and young adults aged 15-39 years from 1990 to 2021: findings from the global burden of disease study 2021. *Front Neurol.* (2025) 16:1535278. 10.3389/fneur.2025.1535278 40144628 PMC11938946

[6] Weterings RP Kessels RP de Leeuw FE Piai V. Cognitive impairment after a stroke in young adults: a systematic review and meta-analysis. *Int J Stroke.* (2023) 18:888–97. 10.1177/17474930231159267 36765436 PMC10507997

[7] Ng AC. Posterior circulation ischaemic stroke. *Am J Med Sci.* (2022) 363:388–98. 10.1016/j.amjms.2021.10.027 35104439

[8] Schneider AM Neuhaus AA Hadley G Balami JS Harston GW DeLuca GC et al. Posterior circulation ischaemic stroke diagnosis and management. *Clin Med.* (2023) 23:219–27. 10.7861/clinmed.2022-0499 37236792 PMC11046504

[9] Xiong J Liu Y Mei L Zhang C Xia J Chen H et al. Vessel wall imaging of vertebrobasilar artery configurations associated with posterior circulation infarction and high-risk atherosclerotic plaques. *Sci Rep.* (2025) 15:14780. 10.1038/s41598-025-96729-6 40295541 PMC12037763

[10] Yu J Zhang S Li ML Ma Y Dong YR Lou M et al. Relationship between the geometry patterns of vertebrobasilar artery and atherosclerosis. *BMC Neurol.* (2018) 18:83. 10.1186/s12883-018-1084-6 29895279 PMC5996488

[11] Gomyo M Tsuchiya K Yokoyama K. Vessel wall imaging of intracranial arteries: fundamentals and clinical applications. *Magn Reson Med Sci.* (2023) 22:447–58. 10.2463/mrms.rev.2021-0140 36328569 PMC10552670

[12] Song JW Wasserman BA. Vessel wall MR imaging of intracranial atherosclerosis. *Cardiovasc Diagn Ther.* (2020) 10:982–93. 10.21037/cdt-20-470 32968655 PMC7487368

[13] Lee HN Ryu CW Yun SJ. Vessel-wall magnetic resonance imaging of intracranial atherosclerotic plaque and ischemic stroke: a systematic review and meta-analysis. *Front Neurol.* (2018) 9:1032. 10.3389/fneur.2018.01032 30559708 PMC6287366

[14] Kang DW Kim DY Kim J Baik SH Jung C Singh N et al. Emerging concept of intracranial arterial diseases: the role of high resolution vessel wall MRI. *J Stroke.* (2024) 26:26–40. 10.5853/jos.2023.02481 38326705 PMC10850450

[15] Zhang J Sun B Wang H Shi H Wen Q Mossa-Basha M et al. Intracranial atherosclerotic plaque features on vessel wall imaging predict first ever and recurrence of stroke: a meta-analysis. *Eur Radiol.* (2025) 35:5017–26. 10.1007/s00330-025-11451-1 39976739 PMC12226209

[16] Sun J Feng XR Feng PY Liu YB Yang HX Yang X et al. HR-MRI findings of intracranial artery stenosis and distribution of atherosclerotic plaques caused by different etiologies. *Neurol Sci.* (2022) 43:5421–30. 10.1007/s10072-022-06132-6 35616814

[17] Qiao Y Zeiler SR Mirbagheri S Leigh R Urrutia V Wityk R et al. Intracranial plaque enhancement in patients with cerebrovascular events on high-spatial-resolution MR images. *Radiology.* (2014) 271:534–42. 10.1148/radiol.13122812 24475850 PMC4263625

[18] Wu F Ma Q Song H Guo X Diniz MA Song SS et al. Differential features of culprit intracranial atherosclerotic lesions: a whole-brain vessel wall imaging study in patients with acute ischemic stroke. *J Am Heart Assoc.* (2018) 7:e009705. 10.1161/JAHA.118.009705 30033434 PMC6201468

[19] Zhu C Tian X Degnan AJ Shi Z Zhang X Chen L et al. Clinical significance of intraplaque hemorrhage in low- and high-grade basilar artery stenosis on high-resolution MRI. *AJNR Am J Neuroradiol.* (2018) 39:1286–92. 10.3174/ajnr.A5676 29794236 PMC6039267

[20] Zhou L Yan Y Du H Ni X Wang G Wang Q. Plaque features and vascular geometry in basilar artery atherosclerosis. *Medicine.* (2020) 99:e19742. 10.1097/MD.0000000000019742 32358348 PMC7440206

[21] Zhou M Yu Y Chen R Liu X Hu Y Ma Z et al. Wall shear stress and its role in atherosclerosis. *Front Cardiovasc Med.* (2023) 10:1083547. 10.3389/fcvm.2023.1083547 37077735 PMC10106633

[22] Mandell DM Mossa-Basha M Qiao Y Hess CP Hui F Matouk C et al. Vessel wall imaging study group of the American society of neuroradiology. intracranial vessel wall MRI: principles and expert consensus recommendations of the American society of neuroradiology. *AJNR Am J Neuroradiol.* (2017) 38:218–29. 10.3174/ajnr.A4893 27469212 PMC7963837

[23] Skarpathiotakis M Mandell DM Swartz RH Tomlinson G Mikulis DJ. Intracranial atherosclerotic plaque enhancement in patients with ischemic stroke. *AJNR Am J Neuroradiol.* (2013) 34:299–304. 10.3174/ajnr.A3209 22859280 PMC7965103

[24] Huang LX Wu XB Liu YA Guo X Ye JS Cai WQ et al. Qualitative and quantitative plaque enhancement on high-resolution vessel wall imaging predicts symptomatic intracranial atherosclerotic stenosis. *Brain Behav.* (2023) 13:e3032. 10.1002/brb3.3032 37128149 PMC10275550

[25] Song JW Pavlou A Burke MP Shou H Atsina KB Xiao J et al. Imaging endpoints of intracranial atherosclerosis using vessel wall MR imaging: a systematic review. *Neuroradiology.* (2021) 63:847–56. 10.1007/s00234-020-02575-w 33029735 PMC8024415

[26] Li Y Guo R Zhang X Yang Y Yan Y Yuan X et al. Effect of dominant vertebral artery angle on basilar artery curvature and plaque. *Quant Imaging Med Surg.* (2023) 13:5748–58. 10.21037/qims-23-74 37711832 PMC10498226

[27] Xu Z Li M Hou Z Lyu J Zhang N Lou X et al. Association between basilar artery configuration and vessel wall features: a prospective high-resolution magnetic resonance imaging study. *BMC Med Imaging.* (2019) 19:99. 10.1186/s12880-019-0388-3 31878890 PMC6933671

[28] Li Y Chen F Yang B Xie S Wang C Guo R et al. Effect of mid-basilar artery angle and plaque characteristics on pontine infarction in patients with basilar artery plaque. *J Atheroscler Thromb.* (2023) 30:182–91. 10.5551/jat.63520 35418542 PMC9925201

[29] Kong P Cui ZY Huang XF Zhang DD Guo RJ Han M. Inflammation and atherosclerosis: signaling pathways and therapeutic intervention. *Signal Transduct Target Ther.* (2022) 7:131. 10.1038/s41392-022-00955-7 35459215 PMC9033871

[30] Di Nubila A Dilella G Simone R Barbieri SS. Vascular extracellular matrix in atherosclerosis. *Int J Mol Sci.* (2024) 25:12017. 10.3390/ijms252212017 39596083 PMC11594217

[31] Song JW Pavlou A Xiao J Kasner SE Fan Z Messé SR. Vessel wall magnetic resonance imaging biomarkers of symptomatic intracranial atherosclerosis: a meta-analysis. *Stroke.* (2021) 52:193–202. 10.1161/STROKEAHA.120.031480 33370193 PMC7773134

[32] Shi Z Li J Zhao M Peng W Meddings Z Jiang T et al. Quantitative histogram analysis on intracranial atherosclerotic plaques: a high-resolution magnetic resonance imaging study. *Stroke.* (2020) 51:2161–9. 10.1161/STROKEAHA.120.029062 32568660 PMC7306260

[33] Guo Y Canton G Baylam Geleri D Balu N Sun J Kharaji M et al. Plaque evolution and vessel wall remodeling of intracranial arteries: a prospective, longitudinal vessel wall MRI study. *J Magn Reson Imaging.* (2024) 60:889–99. 10.1002/jmri.29185 38131254 PMC11192854

[34] Kim BJ Kim HY Jho W Kim YS Koh SH Heo SH et al. Asymptomatic basilar artery plaque distribution and vascular geometry. *J Atheroscler Thromb.* (2019) 26:1007–14. 10.5551/jat.47365 30918163 PMC6845693

[35] Cao S Zhai M He J Wang J Ge T Wu Q et al. Basilar artery curvature increases the risk of posterior circulation infarction occurrence in patients without vertebrobasilar stenosis. *Neurol Sci.* (2023) 44:1273–80. 10.1007/s10072-022-06566-y 36564659 PMC10023619

