# Supplementary material for: Vertebrobasilar artery geometry and quantitative high-resolution vessel wall MRI for stratifying vulnerable plaques in young and middle-aged adults with stroke risk factors
Source: Front Med (Lausanne). 2026 Aug 12;13:1914044. doi: 10.3389/fmed.2026.1914044 (PMC13506843; doi:10.3389/fmed.2026.1914044)
Supplement: Supplementary file 1 [file Table_1.docx]

Supplementary Table 1: MRI acquisition parameters

| Sequence | Orientation / coverage | Imaging technique | TR, ms | TE, ms | TI, ms | Flip angle | FOV, mm | Matrix | Voxel size, mm | Slice thickness, mm | Gap, mm | NEX | Acquisition time |
| --- | --- | --- | --- | --- | --- | --- | --- | --- | --- | --- | --- | --- | --- |
| Axial T1-weighted imaging | Axial whole brain | 2D spin echo | 500 | 8.5 | — | 90° | 230 × 230 | 320 × 256 | 0.72 × 0.90 | 5.0 | 1.0 | 1 | 2 min 15 s |
| Axial T2-weighted imaging | Axial whole brain | 2D turbo spin echo | 4500 | 95 | — | 150° | 230 × 230 | 384 × 288 | 0.60 × 0.80 | 5.0 | 1.0 | 1 | 2 min 30 s |
| Axial T2-FLAIR | Axial whole brain | 2D inversion recovery turbo spin echo | 9000 | 90 | 2500 | 150° | 230 × 230 | 320 × 256 | 0.72 × 0.90 | 5.0 | 1.0 | 1 | 3 min 10 s |
| Diffusion-weighted imaging | Axial whole brain | Single-shot echo-planar imaging | 4000 | 85 | — | 90° | 230 × 230 | 128 × 128 | 1.80 × 1.80 | 5.0 | 1.0 | 2 | 1 min 20 s |
| ADC map | Derived from DWI | b = 0 and 1000 s/mm² | — | — | — | — | — | — | — | — | — | — | Derived |
| 3D TOF-MRA | Axial slab; circle of Willis, vertebral arteries, vertebrobasilar junction, basilar artery | 3D gradient echo TOF | 22 | 3.5 | — | 18° | 200 × 180 | 384 × 320 | 0.52 × 0.56 × 0.60 | 0.60 | 0 | 1 | 4 min 30 s |
| Pre-contrast black-blood T1-weighted HR-VWI | Oblique axial/sagittal coverage of bilateral V4 segments, vertebrobasilar junction, and entire basilar artery | 3D variable-flip-angle turbo spin echo / SPACE | 900 | 15 | — | Variable | 160 × 160 | 320 × 320 | 0.50 × 0.50 × 0.60 | 0.60 | 0 | 1 | 5 min 30 s |
| Black-blood T2-weighted HR-VWI | Same as HR-VWI coverage | 3D variable-flip-angle turbo spin echo / SPACE | 2500 | 90 | — | Variable | 160 × 160 | 320 × 320 | 0.50 × 0.50 × 0.60 | 0.60 | 0 | 1 | 5 min 45 s |
| Proton-density-weighted HR-VWI | Same as HR-VWI coverage | 3D variable-flip-angle turbo spin echo / SPACE | 2000 | 30 | — | Variable | 160 × 160 | 320 × 320 | 0.50 × 0.50 × 0.60 | 0.60 | 0 | 1 | 5 min 20 s |
| Contrast-enhanced T1-weighted HR-VWI | Same as pre-contrast HR-VWI coverage | 3D black-blood T1-weighted SPACE | 900 | 15 | — | Variable | 160 × 160 | 320 × 320 | 0.50 × 0.50 × 0.60 | 0.60 | 0 | 1 | 5 min 30 s |

ADC, apparent diffusion coefficient; DWI, diffusion-weighted imaging; FLAIR, fluid-attenuated inversion recovery; FOV, field of view; HR-VWI, high-resolution vessel wall imaging; MRA, magnetic resonance angiography; NEX, number of excitations; PD, proton density; SPACE, sampling perfection with application-optimized contrasts using different flip-angle evolution; TE, echo time; TI, inversion time; TOF, time-of-flight; TR, repetition time.

Supplementary Table 2: Contrast-enhanced HR-VWI protocol

| Parameter | Description |
| --- | --- |
| Contrast agent | Gadobutrol |
| Dose | 0.1 mmol/kg body weight |
| Route | Intravenous injection |
| Saline flush | 20 mL normal saline |
| Post-contrast delay | Approximately 5 min after contrast administration |
| Post-contrast sequence | Contrast-enhanced black-blood T1-weighted HR-VWI |
| Coverage | Bilateral V4 segments, vertebrobasilar junction, and entire basilar artery |
| Enhancement assessment | Comparison of pre-contrast and post-contrast T1-weighted HR-VWI images |
| Enhancement grading | No obvious enhancement, mild enhancement, or marked enhancement |

Supplementary Table 3: Distribution of primary admission diagnoses in the final analytical cohort

| **Primary admission diagnosis** | **n** | **%** |
| --- | --- | --- |
| Dizziness or vertigo | 52 | 29.9 |
| Headache | 31 | 17.8 |
| Transient ischemic attack or transient focal neurological symptoms | 27 | 15.5 |
| Acute ischemic stroke | 24 | 13.8 |
| Cerebral atherosclerosis or cerebrovascular-risk evaluation | 21 | 12.1 |
| Other neurological disorders | 12 | 6.9 |
| Other non-neurological disorders | 7 | 4.0 |
| **Total** | **174** | **100.0** |

Supplementary Table 4: Interobserver reproducibility of vertebrobasilar geometric and HR-VWI plaque assessments

Supplementary Table 4A: Interobserver reproducibility of quantitative imaging parameters

| **Quantitative parameter** | **Reader 1, mean ± SD** | **Reader 2, mean ± SD** | **ICC (2,1) (95% CI)** | **Interpretation** |
| --- | --- | --- | --- | --- |
| Basilar artery curvature index | 1.164 ± 0.108 | 1.165 ± 0.110 | 0.946 (0.929–0.957) | Excellent |
| Vertebrobasilar junction angle, ° | 61.03 ± 17.40 | 61.60 ± 16.66 | 0.902 (0.865–0.930) | Excellent |
| Basilar artery length, mm | 31.58 ± 4.53 | 31.40 ± 4.46 | 0.948 (0.932–0.961) | Excellent |
| Mean basilar artery diameter, mm | 3.084 ± 0.434 | 3.108 ± 0.447 | 0.922 (0.897–0.941) | Excellent |
| Bilateral vertebral artery diameter difference, mm | 0.640 ± 0.322 | 0.630 ± 0.322 | 0.898 (0.865–0.925) | Good |
| Maximum basilar artery deviation, mm | 4.886 ± 2.134 | 4.912 ± 2.094 | 0.891 (0.856–0.917) | Good |
| Lumen area, mm² | 9.403 ± 2.477 | 9.323 ± 2.470 | 0.890 (0.850–0.920) | Good |
| Total vessel area, mm² | 21.258 ± 4.034 | 21.027 ± 3.836 | 0.886 (0.853–0.913) | Good |
| Wall area, mm² | 12.279 ± 3.356 | 12.363 ± 3.485 | 0.923 (0.897–0.943) | Excellent |
| Plaque burden, % | 53.636 ± 10.509 | 53.463 ± 10.673 | 0.889 (0.852–0.916) | Good |
| Percentage area stenosis, % | 34.445 ± 13.098 | 34.964 ± 13.934 | 0.877 (0.835–0.907) | Good |
| Remodeling index | 1.068 ± 0.140 | 1.077 ± 0.136 | 0.890 (0.859–0.918) | Good |

Supplementary Table 4B: Interobserver agreement for qualitative imaging features

| **Qualitative feature** | **Reader 1 distribution, n (%)** | **Reader 2 distribution, n (%)** | **Overall agreement, %** | **Agreement statistic (95% CI)** | **Interpretation** |
| --- | --- | --- | --- | --- | --- |
| Plaque location | V4 vertebral artery: 78 (44.8%); basilar artery: 96 (55.2%) | V4 vertebral artery: 78 (44.8%); basilar artery: 96 (55.2%) | 96.6 | κ = 0.930 (0.865–0.977) | Almost perfect |
| Plaque distribution | Concentric: 55 (31.6%); eccentric: 119 (68.4%) | Concentric: 55 (31.6%); eccentric: 119 (68.4%) | 94.3 | κ = 0.867 (0.779–0.942) | Almost perfect |
| Plaque-enhancement grade | Grade 0: 64 (36.8%); grade 1: 67 (38.5%); grade 2: 43 (24.7%) | Grade 0: 57 (32.8%); grade 1: 73 (42.0%); grade 2: 44 (25.3%) | 92.0 | Weighted κ = 0.932 (0.893–0.966) | Almost perfect |
| Plaque surface morphology | Regular: 154 (88.5%); irregular: 20 (11.5%) | Regular: 153 (87.9%); irregular: 21 (12.1%) | 97.1 | κ = 0.862 (0.725–0.960) | Almost perfect |
| Intraplaque hemorrhage | Absent: 161 (92.5%); present: 13 (7.5%) | Absent: 161 (92.5%); present: 13 (7.5%) | 97.7 | κ = 0.834 (0.635–0.965) | Almost perfect |
| Intraplaque signal pattern | Homogeneous: 147 (84.5%); heterogeneous/complex: 27 (15.5%) | Homogeneous: 146 (83.9%); heterogeneous/complex: 28 (16.1%) | 96.0 | κ = 0.849 (0.724–0.955) | Almost perfect |
| Remodeling category | Negative: 34 (19.5%); no obvious remodeling: 56 (32.2%); positive: 84 (48.3%) | Negative: 36 (20.7%); no obvious remodeling: 59 (33.9%); positive: 79 (45.4%) | 92.5 | Weighted κ = 0.938 (0.903–0.970) | Almost perfect |
| Basilar artery bending grade | None: 48 (27.6%); mild: 69 (39.7%); moderate: 42 (24.1%); severe: 15 (8.6%) | None: 47 (27.0%); mild: 68 (39.1%); moderate: 41 (23.6%); severe: 18 (10.3%) | 90.8 | Weighted κ = 0.947 (0.918–0.972) | Almost perfect |
| Vertebral artery dominance | No obvious dominance: 41 (23.6%); left: 72 (41.4%); right: 61 (35.1%) | No obvious dominance: 41 (23.6%); left: 72 (41.4%); right: 61 (35.1%) | 93.1 | κ = 0.894 (0.834–0.947) | Almost perfect |
| HR-VWI-defined vulnerable plaque phenotype | Non-vulnerable: 105 (60.3%); vulnerable: 69 (39.7%) | Non-vulnerable: 105 (60.3%); vulnerable: 69 (39.7%) | 95.4 | κ = 0.904 (0.839–0.961) | Almost perfect |

Values represent independent assessments by two radiologists before consensus resolution. Intraclass correlation coefficients were calculated using a two-way random-effects, absolute-agreement, single-measurement model [ICC(2,1)]. Cohen’s kappa was used for binary and nominal variables, whereas quadratic-weighted kappa was used for ordinal variables. ICC values were interpreted as poor (<0.50), moderate (0.50–0.75), good (0.75–0.90), or excellent (>0.90). Kappa values were interpreted as slight (≤0.20), fair (0.21–0.40), moderate (0.41–0.60), substantial (0.61–0.80), or almost perfect (0.81–1.00). BA, basilar artery; CI, confidence interval; HR-VWI, high-resolution vessel-wall imaging; ICC, intraclass correlation coefficient; SD, standard deviation; V4, intracranial vertebral artery segment.

Supplementary Table 5: Univariable screening, collinearity diagnostics, and variable-selection results for the HR-VWI-defined vulnerable plaque phenotype

Supplementary Table 5A: Univariable screening and initial collinearity diagnostics

| **Candidate predictor** | **Reporting unit** | **Univariable OR (95% CI)** | **P value** | **Eligible for entry** | **Tolerance** | **VIF** | **Modeling decision** |
| --- | --- | --- | --- | --- | --- | --- | --- |
| Age | Per 5-year increase | 1.26 (1.02–1.55) | 0.032 | Yes; forced | 0.847 | 1.18 | Retained as a prespecified confounder |
| Body mass index | Per 1-kg/m² increase | 1.13 (1.02–1.25) | 0.021 | Yes; forced | 0.820 | 1.22 | Retained as a prespecified confounder |
| Basilar artery curvature index | Per 0.1-unit increase | 1.76 (1.28–2.42) | <0.001 | Yes | 0.415 | 2.41 | Removed during backward elimination, P = 0.372 |
| Vertebrobasilar junction angle | Per 10° increase | 1.35 (1.12–1.63) | 0.002 | Yes | 0.568 | 1.76 | Removed during backward elimination, P = 0.241 |
| Basilar artery length | Per 1-mm increase | 1.07 (1.00–1.15) | 0.051 | Yes | 0.610 | 1.64 | Removed during backward elimination, P = 0.614 |
| Mean basilar artery diameter | Per 1-mm increase | 1.62 (0.80–3.28) | 0.180 | No | — | — | Not entered because univariable P ≥0.10 |
| Bilateral vertebral artery diameter difference | Per 0.1-mm increase | 1.17 (1.06–1.29) | 0.002 | Yes | 0.529 | 1.89 | Removed during backward elimination, P = 0.118 |
| Maximum basilar artery deviation | Per 1-mm increase | 1.29 (1.11–1.50) | <0.001 | Yes | 0.420 | 2.38 | Retained in the final model |
| Plaque burden | Per 1% increase | 1.12 (1.07–1.18) | <0.001 | Yes | 0.212 | 4.72 | Retained after assessment of related area variables |
| Wall area | Per 1-mm² increase | 1.26 (1.12–1.41) | <0.001 | Yes | 0.146 | 6.84 | Excluded before backward elimination because of collinearity |
| Percentage area stenosis | Per 1% increase | 1.05 (1.02–1.08) | <0.001 | Yes | 0.340 | 2.94 | Retained in the final model |
| Remodeling index | Per 0.1-unit increase | 1.68 (1.29–2.19) | <0.001 | Yes | 0.461 | 2.17 | Retained in the final model |
| Lumen area | Per 1-mm² increase | 0.89 (0.79–1.00) | 0.045 | Yes | 0.178 | 5.63 | Excluded before backward elimination because of collinearity |
| Total vessel area | Per 1-mm² increase | 1.07 (0.99–1.14) | 0.073 | Yes | 0.159 | 6.29 | Excluded before backward elimination because of collinearity |

Supplementary Table 5B: Collinearity diagnostics for predictors retained in the final model

| **Retained predictor** | **Reporting unit** | **Tolerance** | **VIF** | **Adjusted OR (95% CI)** | **P value** |
| --- | --- | --- | --- | --- | --- |
| Age | Per 5-year increase | 0.901 | 1.11 | 1.15 (0.93–1.43) | 0.203 |
| Body mass index | Per 1-kg/m² increase | 0.917 | 1.09 | 1.07 (0.95–1.21) | 0.273 |
| Plaque burden | Per 1% increase | 0.481 | 2.08 | 1.08 (1.03–1.13) | 0.001 |
| Percentage area stenosis | Per 1% increase | 0.581 | 1.72 | 1.03 (1.00–1.06) | 0.047 |
| Remodeling index | Per 0.1-unit increase | 0.719 | 1.39 | 1.82 (1.01–3.29) | 0.047 |
| Maximum basilar artery deviation | Per 1-mm increase | 0.847 | 1.18 | 1.20 (1.01–1.43) | 0.040 |

Univariable odds ratios were estimated using binary logistic regression, with the HR-VWI-defined vulnerable plaque phenotype as the dependent variable. Predictors with a univariable P value <0.10 were eligible for multivariable entry. Age and body mass index were prespecified potential confounders and were forced into the multivariable model irrespective of statistical significance. Collinearity was considered potentially important when tolerance was ≤0.20 or VIF was ≥5. Wall area, lumen area, and total vessel area were excluded before backward elimination because of collinearity with plaque burden and related area-derived measurements. The remaining eligible variables were evaluated using backward likelihood-ratio elimination, with an entry criterion of P <0.05 and a removal criterion of P >0.10. Age and body mass index were protected from elimination. All predictors retained in the final model had tolerance values >0.20 and VIF values <5. CI, confidence interval; HR-VWI, high-resolution vessel-wall imaging; OR, odds ratio; VIF, variance inflation factor.

Supplementary Table 6A: Sensitivity analysis using the largest measurable plaque irrespective of vulnerability status

| **Predictor** | **Reporting unit** | **Primary adjusted OR (95% CI)** | **Sensitivity adjusted OR (95% CI)** | **P value** |
| --- | --- | --- | --- | --- |
| Age | Per 5-year increase | 1.15 (0.93–1.43) | 1.14 (0.92–1.41) | 0.226 |
| Body mass index | Per 1-kg/m² increase | 1.07 (0.95–1.21) | 1.06 (0.94–1.20) | 0.319 |
| Plaque burden | Per 1% increase | 1.08 (1.03–1.13) | 1.07 (1.03–1.12) | 0.002 |
| Percentage area stenosis | Per 1% increase | 1.03 (1.00–1.06) | 1.03 (1.00–1.06) | 0.041 |
| Remodeling index | Per 0.1-unit increase | 1.82 (1.01–3.29) | 1.76 (1.02–3.04) | 0.043 |
| Maximum basilar artery deviation | Per 1-mm increase | 1.20 (1.01–1.43) | 1.18 (1.01–1.39) | 0.037 |

Supplementary Table 6B: Apparent discrimination of the models in the uniform largest-plaque sensitivity analysis

| **Model** | **Primary AUC (95% CI)** | **Sensitivity AUC (95% CI)** | **Sensitivity** | **Specificity** |
| --- | --- | --- | --- | --- |
| Age and BMI | 0.637 (0.554–0.720) | 0.637 (0.554–0.720) | 66.67% | 58.10% |
| Age and BMI plus geometry | 0.774 (0.705–0.843) | 0.774 (0.705–0.843) | 72.46% | 72.38% |
| Age and BMI plus quantitative HR-VWI | 0.883 (0.832–0.934) | 0.872 (0.817–0.927) | 84.06% | 77.14% |
| Age and BMI plus combined imaging | 0.903 (0.858–0.948) | 0.894 (0.846–0.942) | 76.81% | 88.57% |

## Supplementary Table 6C: Pairwise DeLong comparisons in the uniform largest-plaque sensitivity analysis

| **Comparison** | **ΔAUC** | **P value** |
| --- | --- | --- |
| Geometry model vs. clinical model | 0.137 | 0.003 |
| Quantitative HR-VWI model vs. clinical model | 0.235 | <0.001 |
| Combined imaging model vs. clinical model | 0.257 | <0.001 |
| Combined imaging vs. quantitative HR-VWI model | 0.022 | 0.148 |
